# The Long-Lasting Influenza: The Impact of Fetal Stress During the 1918 Influenza Pandemic on Socioeconomic Attainment and Health in Sweden, 1968–2012

**DOI:** 10.1007/s13524-019-00799-x

**Published:** 2019-07-19

**Authors:** Jonas Helgertz, Tommy Bengtsson

**Affiliations:** 1 Centre for Economic Demography (CED) and Department of Economic History, Lund University, Box 7083, 220 07 Lund, Sweden; 2 Minnesota Population Center, University of Minnesota, 50 Willey Hall, 225 19th Avenue South, Minneapolis, MN 55455 USA; 3 IZA, Institute of Labor Economics, Schaumburg-Lippe-Strasse 5-9, 53113 Bonn, Germany; 4 CEPR, Centre for Economic Policy Research, 33 Great Sutton Street, London, EC1V 0DX UK

**Keywords:** Fetal origins, Spanish influenza pandemic, Sweden, Health and socioeconomic outcomes, Longitudinal data

## Abstract

The 1918 influenza pandemic had not only a massive instant death toll but also lasting effects on its survivors. Several studies have shown that children born in 1919, and thus exposed to the H1N1 virus *in utero*, experienced worse health and socioeconomic outcomes in older ages than surrounding birth cohorts. This study combines several sources of contemporary statistics with full-population individual-level data for Sweden during 1968–2012 to examine the influence of fetal exposure to the Spanish flu on health, adulthood income, and occupational attainment. For both men and women, fetal exposure resulted in higher morbidity in ages 54–87, as measured by hospitalization. For males, exposure during the second trimester also affected mortality in cancer and heart disease. Overall, the effects on all-cause mortality were modest, with about three months shorter remaining life expectancy for the cohorts exposed during the second trimester. For socioeconomic outcomes, results fail to provide consistent evidence supporting any long-term consequences of fetal exposure. We conclude that although the immediate health effects of exposure to the 1918 pandemic were huge, the long-term effects were modest in size.

**Electronic supplementary material:**

The online version of this article (10.1007/s13524-019-00799-x) contains supplementary material, which is available to authorized users.

## Introduction

On the centennial anniversary of the 1918 influenza pandemic, commonly known as *the Spanish flu*, its relevance among the public and the scientific community is still high. Recalling its massive death toll, reported by some sources to have been at least 50 million individuals (Institute of Medicine 2005), the world in 2009 again braced for the impact of an influenza pandemic with considerable similarities to the Spanish flu, both resulting from an antigenic shift in the H1N1 virus. As a result, governments across the world invested in vaccination campaigns and issued guidelines to limit the consequences of a pandemic. The fears were linked both to its potentially huge immediate effects in terms of deaths and to the possible long-lasting consequences among those surviving the disease, including those exposed *in utero*. This article examines the long-term consequences of fetal exposure to the 1918 influenza pandemic, using uniquely detailed Swedish longitudinal individual-level data for the period 1968–2012, allowing for the examination of a range of health and socioeconomic outcomes.

The 1918 influenza pandemic has been used as a quasi-experiment in several previous studies to analyze the causal effects of a fetal insult. Studies on the United States have shown that children born in 1919—and thus exposed to the H1N1 virus *in utero*—experienced worse health (Almond and Mazumder 2005) and higher mortality in older ages (Fletcher 2018; Mazumder et al. 2010; Myrskylä et al. 2013). Those exposed also had lower educational attainment, higher rates of physical disability, lower income attainment, lower socioeconomic status (SES), and a higher dependence on social welfare than surrounding birth cohorts (Almond 2006). Similar results have been found for Switzerland (Neelsen and Stratmann 2012), Brazil (Nelson 2010), Taiwan (Lin and Liu 2014) and Sweden (Richter and Robling 2015). Other studies found much weaker effects, if any at all. Using data from the Human Mortality Database, a study of 24 countries found that overall mortality did not differ systematically for *in utero*–exposed cohorts relative to surrounding cohorts (Cohen et al. 2010). Another study, using census data for 117 countries, found no consistent long-term effects of influenza exposure on education, employment, or disability outcomes and concluded that the evidence on long-term economic effects of the Spanish flu is likely a consequence of publication bias (Vollmer and Wójcik 2017). Brown and Thomas (2013) suggested that the strong health and socioeconomic effects found in early studies on the United States were driven by a shift in the socioeconomic composition of the 1919 birth cohort rather than being causally linked to fetal exposure to the Spanish flu. Beach et al. (2018), however, questioned this claim and found effects also after controlling for the individual’s socioeconomic origin, again with U.S. data.

At a first glance, the 1918 pandemic looks like an ideal quasi-experiment for studying the fetal origins hypothesis. It was severe, unexpected, and spread worldwide in a short period of time with no effective means available to prevent its transmission. Furthermore, its short duration makes it possible—with access to detailed data on time of birth—to effectively distinguish between prenatal and postnatal exposure. What makes it potentially problematic from a quasi-experimental perspective, however, is that it took place during World War I (WWI), when many other factors that are known to influence fetal development operated as well. During wartime, married couples also often became separated, thus postponing births and leading to temporary declines in fertility. If the change in fertility was correlated with any characteristic known to also influence the outcome of interest, this will introduce biased estimates. For example, as shown for the United States, because the enlistment to WWI at the time of the pandemic had a social gradient, the socioeconomic composition of newborns changed accordingly (Brown and Thomas 2013). If children born to parents with low SES are less healthy and will inherit part of their parents’ socioeconomic disadvantage, it becomes difficult to estimate the causal effect of the pandemic. Indeed, evidence from the United States suggests that failing to take this into account overestimates the effects of fetal programming (Brown and Thomas 2013). Another study for the United States, linking microdata from the 1920 and 1930 censuses to WWII enlistment records and city-level influenza data, however, contradicts this argument; this study found that in the absence of the pandemic, the 1919 birth cohort would have been more likely to graduate from high school—an result that was largely unaffected by including controls for parental characteristics (Beach et al. 2018).

These issues are by no means are restricted to studies on the United States but instead characterize most empirical analyses on the consequences of fetal exposure to the Spanish flu. One important exception is a study of Taiwan that not only had individual-level information on parents’ characteristics but also involved a context not involved in WWI (Lin and Liu 2014). As a result, the study was able to examine the consequences of fetal exposure to the 1918 influenza pandemic net of such selection mechanisms. The study found that neither source of selection was strong enough to counteract the effect of the flu, influencing both health and cognitive outcomes. In comparing the long-term consequences of the Spanish influenza pandemic across contexts, it should, however, be borne in mind that Taiwan was characterized by a very different disease environment compared with more-developed countries at that time, with a considerably higher infant mortality rate and presence of cholera and malaria. As a consequence, as the authors noted, the study of Taiwan is of higher relevance to present-day sub-Saharan Africa and other developing countries rather than to developed countries (Lin and Liu 2014:153).

In this article, we study the long-term effects of fetal exposure to the 1918 pandemic in Sweden, a country affected as severely as any other. When the pandemic peaked in October–November, mortality was more than twice as high as during a typical year. We use longitudinal individual-level data to examine the birth cohorts 1914–1925 in the period 1968–2012. Through detailed data on time and place of birth, we are able to distinguish between fetal and child exposure, also exploiting differences in influenza timing and intensity between geographical regions. Our focus is on hospitalization and mortality by cause of death as well as on income and occupational attainment. The analysis of socioeconomic outcomes adds to previous work on the effects of the pandemic in Sweden on income and occupation attainment using individual-level data, which have shown conflicting results depending on sample and model specification (Richter and Robling 2013, 2015). Our study also adds to our present knowledge concerning health effects of the flu by analyzing hospitalization data by separate disease type, serving as a complement to the mortality analysis.

Because we use data from 1968 and onward, our study shares several of the problems of previous studies in isolating the effects of the pandemic. Arguably of greatest importance is that we do not have access to information on individual-level parental background information, such as SES. This is an obvious weakness compared with the study of the United States by Brown and Thomas (2013), who used information from the 1930 census to measure parental occupation early in life, and the study of Taiwan by Lin and Liu (2014). Aggregated annual statistics on the occupation of fathers of newborns in Sweden, however, indicates that the socioeconomic composition changed only marginally. Furthermore, as opposed to the situation in the United States, this change was not exclusive to the birth cohorts who were exposed while *in utero*. Instead, the share of newborns to well-off fathers was about 1 percentage point higher in 1918 and 1919 compared with the years before and after, thus affecting other cohorts in addition to those exposed while *in utero*. This is very important because it would otherwise be impossible to separate the effects of the pandemic from changes in SES. Survival selection is also likely to be quite marginal. Although stillbirths increased nontrivially in relative terms, the number of stillborn children was still very small, increasing by only around 1 stillbirth for each 100 live-born babies. A similar story applies to infant deaths credibly attributable to the influenza during the peak months of the pandemic. Furthermore, given that infant mortality in general increased only marginally and both the sex ratio at birth and birth weight remained stable, we believe that survival selection is negligible.

We find evidence of fetal programming on hospitalization and mortality in cancer and cardiovascular diseases among males exposed to the flu during the second trimester. For females exposed during the third trimester of pregnancy, we find that influenza exposure increased the risk of hospitalization but had no effects on mortality. For income and occupational attainment, we find no conclusive results regarding the relationship between influenza exposure and later-life outcomes. We conclude that although the immediate health effects of exposure to the Spanish flu were considerable, the long-term effects were small, both for males and for females.

## Theory

The growth of the fetus rapidly accelerates during the early phase of pregnancy, slowing down after 20 weeks. Particularly accentuated during this initial stage is the development of the child’s body structure and certain organs and systems, such as the brain (Brown and Derkits 2010), the heart (Hoet and Hansen 1999; Thornburg 2001), and the metabolic system (Harding and Gluckman 2001). This is also the period when most miscarriages (about 80 %) and birth defects occur. Disturbances during this period are associated with a range of diseases, including schizophrenia, coronary heart disease, diabetes, and breast and testicular cancers (Kuh and Ben-Shlomo 2004). Instead, the kidneys and lungs are primarily developed during the last stages of pregnancy (Lackland 2005; Thornburg 2001), which is why disturbances during this period may cause renal disease and impaired lung functions.

Because the virus of both the pandemic (Zou 2006) and the seasonal influenza (Irving et al. 2000) rarely is transmitted across the placenta, it is more likely to affect the fetus through maternal fever and inflammation (Fell et al. 2017). Immunological responses to influenza can also influence placental functioning and cause preterm birth, and have also been shown to be associated with defects of the central nervous system (CNS) as well as increased risks for schizophrenia and child leukemia (Nelson 2010). A recent review of maternal influenza and birth outcomes, however, found the evidence to be limited (Fell et al. 2017). Still, evidence suggests that secondary pneumonia, which was common during the 1918 pandemic, can contribute to fetal deaths (Harris 1919).

Prenatal disturbances have different consequences depending on the severity of the insult as well as the stage of fetal development. If the insult occurs during the first part of the pregnancy, it is likely to lead miscarriages and spontaneous abortions, which should influence the sex ratio at birth given that male fetuses are more vulnerable than female fetuses (see Quaranta 2013). Disturbances during the latter part of the pregnancy could instead lead to an increase in stillbirths and preterm births.

Low birth weight, one of the most common indicators of exposure to adversity *in utero*, has been shown to be associated with high blood pressure and an increased risk of heart disease at adult ages (Barker 1994; Christensen 2019; Huxley et al. 2002; Risnes et al. 2011), schizophrenia (Brown and Derkits 2010), and diabetes (Hales et al. 1991; Harding and Gluckman 2001). Not established, however, is whether low birth weight primarily is the result of early (Smith et al. 2002) or late (Strauss and Dietz 1999) fetal adversity. Evidence suggests, however, that high birth weight is programmed through maternal weight gain during early pregnancy, during the first and second trimesters (Brown and Avery, 2012; Neufeld et al. 2004). Somewhat counterintuitively, high birth weight is associated with increased incidences of breast cancer (Risnes et al. 2011) and prostate cancer (Risnes et al. 2011) later in life. Thus, the relationship between birth weight and diseases in childhood and later in life appears to be U-shaped.

Low birth weight has been shown to be linked to negative effects on educational and labor market outcomes (Currie and Moretti 2007; Johnson and Schoeni 2007; Royer 2009). Those with low birth weight perform worse in school, are less likely to be employed in adulthood, and earn less in adulthood than those with normal birth weight (Case et al. 2005; Currie and Hyson 1999; Palloni et al. 2009). Other studies, however, found only small effects of birth weight. A study of Norway using register data, for example, found that a 10 % lower birth weight—a sizable difference from the norm—is associated with only 1.2 % lower IQ for males, 0.3 % shorter height, and 0.9 % lower earnings (Black et al. 2007).

After birth, the development of cells and organs continues, gradually slowing and completing around the time when the individual is about 20 years of age. For example, the lungs keep growing after birth and until about 8 years of age, a period during which new alveolar sacs are also generated (Broman 1923). Because the child remains vulnerable to adverse conditions after birth, exposure to the Spanish flu might have caused damage not only during the fetal stage but also during the first months of life. Streptococcal infections in early childhood, for example, are known to be associated with rheumatic heart disease (Jones 1956), whereas respiratory infections have been suggested to impair lung function (Barker et al. 1991; Bengtsson and Lindström 2003) and are also associated with diseases such as cancer and diabetes in older ages (Finch and Crimmins 2004; see Quaranta 2013:162–168). Infections in general during infancy and beyond may also cause inflammation in atherosclerosis, which is a risk factor for a variety of later-life diseases, including cardiovascular diseases, diabetes, and some forms of cancer (Finch 2009; Libby 2002). Because the child remains sensitive to environmental insults into the first years of life, it is important to analyze the timing of the exposure of the Spanish flu in detail to distinguish between prenatal and postnatal programming.

## The Use of the 1918 Influenza as a Quasi-Experiment in Sweden

The ability to use the 1918 influenza pandemic as a quasi-experiment to test the fetal origins hypothesis is based on the fulfillment a number of assertions. First, the event should be significant enough to allow its effects to be traced. Second, the event should not be anticipated. Third, it should affect only fetuses among those exposed. Fourth, no other factor affecting fetal development should take place at the same time as the pandemic.

The first assertions are easy to confirm for Sweden. A distinguishing feature of the 1918 influenza was that the first wave occurred unusually early, in the summer; the influenza period in Sweden normally occurs during the winter. Furthermore, because of the low initial death toll, the arrival of the influenza was hardly recognized. The second wave also started early, in September, and peaked in October–November 1918, but resulted in dramatically increased mortality, with about 160 deaths per 100,000 individuals. The pandemic again resurged during the late spring of 1919 as a comparably mild wave of the flu, after which mortality returned to normal levels. In Sweden, the 1918 pandemic claimed at least 34,000 deaths in a population of 5.8 million. As in other parts of the world, the influenza spread across the country in a short period. Certain differences in timing and intensity, however, can still be observed (see Fig. A1, online appendix, for a subset of Swedish counties). And although certain preventive actions were taken, the spread of the disease nevertheless remained impossible to stop (Åman 1990).

According to official sources, only 9 % of the entire Swedish population was reported to have been sick from the flu, with the figure for the comparatively densely populated capital city of Stockholm being as low as 1.43 % (Åman 1990:58). Contemporary authorities, however, deemed the officially reported morbidity figures as far too low, something that applied to the United States as well (Åman 1990; Crosby 1989). Studies of local workplaces, where the population at risk is precisely defined and the recording of illness is of high quality, corroborate this claim (Alling 1919; Gibson 1919; Widstrand 1918). At *Höganäsverket*, a coal mine and factory located in the southwest of Sweden, 61 % of all males and 50 % of all females, families included, were reported by the company physician as showing clear symptoms (Alling 1919). Among men aged 15–20, the sickness rate was as high as 81 %. Similar figures were reported at other workplaces in Sweden, shown in Fig. A2 (online appendix). Furthermore, white- and blue-collar workers at *Höganäsverket* were reported to have the same incidence rate, a pattern representative of Sweden as a whole (Alling 1919; Åman 1990; Gibson, 1919). Among mining workers, 56 % were reported to have been on sick leave, for an average of 14 days (Alling 1919; *Höganäsverkets arkiv*^1919^). One-third of those who were ill were reported suffering badly from the disease. Although the proportion of persons with clinical signs of the disease during a normal influenza outbreak is 30 % to 60 % (Gagnon et al. 2013), roughly 75 % of the population showed clear symptoms of the 1918 influenza in Sweden (Åman 1990:58–59). Bengtsson et al. (2018) found that influenza affected all socioeconomic groups, with slightly lower mortality for farmers but no clear social gradient in mortality.

Despite the severe underreporting of persons having influenza symptoms in official sources, the mortality and morbidity rates followed an almost identical time pattern (see Fig. A3, online appendix). By the end of 1918, some 83 % of those ever reported to be ill to the medical authorities had contracted the disease, and 80 % of the deaths that would be attributed to the pandemic had occurred. Thereafter, the death rate fell, only to resurge somewhat during the third wave in March–April 1919. Thus, as elsewhere, the 1918 influenza pandemic was very short and intensive in Sweden. Furthermore, the fact that the spikes in morbidity and mortality virtually coincided implies that the monthly mortality development can be used both as a measure of the intensity and the timing of morbidity.^1^

Turning to the third assertion—that the pandemic affected only fetuses—we initially consider the particular age pattern of the 1918 pandemic. Although most influenza outbreaks do not change the typical U-shaped pattern of age-specific mortality, the 1918 pandemic elevated mortality in early to middle adulthood. The result was the now familiarly labeled W-shaped mortality pattern reported from all over the world (see Fig. A4, online appendix).

Several explanations have been proposed to account for the disproportionately high mortality among young adults, ranging from partial immunity among older adults who had experienced the Russian flu of 1889–1890 and vulnerability among young adults having tuberculosis, to overactive immune systems among young adults or T-cell deregulation (see Gagnon et al. 2013). The notion of a W-shaped mortality pattern, however, also implies that infants suffered disproportionately from the flu. If so, surviving individuals belonging to the comparison group, who were exposed postnatally, could be characterized by scarring given that infections in first months of life are known to affect both health and socioeconomic outcomes later in life (Bengtsson and Lindström 2000, 2003; see Quaranta 2013 for an overview). Conversely, fetal exposure could also result in positive selection if only the more robust children survive.

A comparison of all-cause mortality in 1918 with that in 1917, however, casts some doubts about whether the age-specific mortality pattern of the 1918 influenza should be characterized as W-shaped. Although infant mortality was slightly higher during the fall of 1918 compared with the same months the year before, mortality at an annual level was in fact slightly lower (see Fig. A5, online appendix). The same pattern was observed in Denmark and Finland; in the United States, mortality was slightly higher in the fall of 1918 than in the year before. Furthermore, mortality among the elderly was not higher in 1918 than in the year before, lending further support to the claim that labeling the mortality age pattern of the 1918 influenza pandemic as W-shaped in Sweden is misleading, a conclusion also supported by hospital records (Holtenius and Gillman 2014). What is certain is that mortality among young adults was particularly elevated. Contemporary sources highlighted that pregnant women were particularly vulnerable, but mortality was still lower for women than for men (Almond 2006:681; Åman 1990).

Finally, we turn to the fourth assertion—that there should not be any omitted variable(s) concurrently affecting exposure and outcome, such as variables related to changes in the socioeconomic position of parents to children born during the influenza (Brown and Thomas 2013) or other forms of selective fertility. Although Sweden did not participate in WWI, more men than usual were nonetheless recruited to the army. The magnitude of this recruitment, though, was comparatively modest, and unlike in the United States, recruits were not excluded because they were poor. As a result, we do not expect any changes in the social composition of newborn children due to military enlistment. A related issue is whether conceptions during the pandemic, which decreased (Boberg-Fazlic et al. 2017) as reflected in the number of births during nine months after September, October, and November 1918 (Fig. A6, online appendix), altered the socioeconomic or health composition of newborns.

Although we do not have individual-level data on the socioeconomic origin of individuals, we do have annual occupation data from aggregate national statistics on fathers of newborns for the years 1911–1920 and 1924–1930 (Befolkningsrörelsen 1911–1930). Occupations are reported in 63 categories, which we use to distinguish between different social groups.^2^ For example, in agriculture (the largest sector at the time), we can distinguish between landowners and workers. Similarly, factory owners are separately reported within the industrial sector, and higher civil servants are separately reported in the service sector. Making the distinction in the trading sector is slightly more difficult because of uncertainty regarding whether the group of merchants belongs to one group or the other. However, the overall pattern of the social gradient of the newborns remains the same regardless of the classification of merchants.

Figure A7 (online appendix) shows that the proportion of newborn children to well-off fathers indeed increased gradually from 1911 to 1919; the share in 1918 and 1919 was a bit more than 1 percentage point higher than in the surrounding years. Because data on parental SES are lacking for 1921–1923, the figure also shows the share of fathers under the age of 30, who are expected to disproportionately be of low SES. Indeed, the series largely mirror each other; an increasing share of young fathers is correlated with a declining share of high-SES fathers. The data also suggest that the birth cohorts of 1918 and 1919 are similar in terms of the share of high-SES fathers but differ modestly in that the 1919 cohort has younger fathers. Thus, the declining births nine months after the influenza months seem to have been the result of the (healthier) younger being more able to or choosing to conceive rather than due to a strong process of fetal health selection (as demonstrated in the remainder of this section). Although it is difficult to fully assess the socioeconomic selection mechanisms to which the *in utero*–exposed cohorts were subjected, the shift in the socioeconomic background compared with those born five years before and after suggests a bias in the opposite direction compared with the United States; this bias also extends to those exposed during the first 12 months of life. Thus, compared with the situation in the United States, where the direction of the socioeconomic bias would lead to the overestimation of the effects of the Spanish flu, the Swedish context would, if anything, result in an underestimation of the effects because of positive selection on socioeconomic background, at least when socioeconomic outcomes are considered. More importantly, because the proportion of children born to well-off parents was high over a two-year period, we should be better able to distinguish this shift in socioeconomic status from the effects of the pandemic on the fetus, which was concentrated in a three-month period. Again, the Taiwanese context that Lin and Liu (2014) examined is plausibly a case study wherein the influence of selection mechanisms linked to parental characteristics are minimized, largely confirming Almond’s (2006) conclusions that the Spanish influenza pandemic indeed had long-term causal effects.

The intergenerational transmission of SES in Sweden has been strong for cohorts born throughout the twentieth century (Dribe et al. 2015), an important factor to consider when examining the link between Spanish flu exposure and adulthood socioeconomic outcomes in light of the changing socioeconomic composition of fathers. Similarly, the positive socioeconomic selection experienced by the *in utero*–exposed cohorts in particular could have repercussions for the other set of outcomes examined in this study: the individual’s health. The existence of a socioeconomic health gradient is taken for granted in contemporary Western societies, with a substantial penalty experienced by the socioeconomically disadvantaged. Thus, given the strong intergenerational correlation of SES, it follows that a part of the health advantage enjoyed by high-SES individuals would be inherited. Several studies, however, have concluded that the magnitude of this relationship is a relatively recent one and that the assumption that the socioeconomic gradient has existed for a very long time is due to a dominance of studies taking a period rather than a cohort approach. Two studies using data for the United States showed that mortality differences by educational attainment for white men and women remained modest at least until the late-1920 birth cohorts, after which they diverged considerably (Lauderdale 2001; Masters et al. 2012). Both findings are consistent with an increasingly robust cohort morbidity phenotype: later-born cohorts experienced fewer and less harsh insults throughout life and, in particular, during early life.

Turning to indicators of fetal selection on health, we initially examine birth weight. A study of births in Sweden between 1918 and 1922 found very small changes in birth weight from one year to the next (Abolins 1962). For example, the birth weight in 1919 was, on average, 17 and 14 g higher for girls and boys, respectively, than in the preceding year. Although birth weights in Norway during the war were also stable, they declined in France, Holland, Italy, Austria, Germany, and Russia, but with the timing of the decline consistently appearing to be unrelated to the Spanish flu pandemic. For example, whereas the decline started in 1915/1916 in Berlin, Würzberg, and Marburg, it started in 1918 in Basel (Abolins 1962). Furthermore, the decline was relatively marginal at 60–80 g. The fact that neither the timing nor the spatial distribution of changes in birth weight followed the influenza pandemic lends no support to the hypothesis that the pandemic caused low birth weight.

**Fig. 1:**
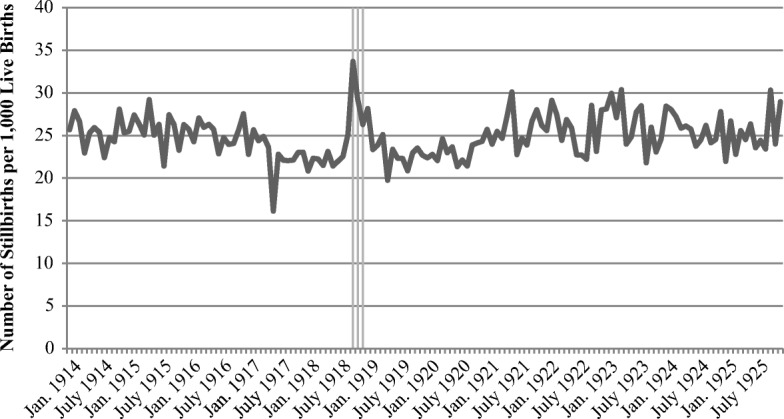
Stillbirths per 1,000 live births, by month. Sweden, 1914–1925. The shaded vertical bars represents the period October–December 1918. *Source:* Sveriges Officiella Statistik: Befolkningsrörelsen 1914–1925.

Figure 1 shows monthly data on stillbirths expressed as a share of every 1,000 live births obtained from official aggregate Swedish statistics during the period 1914–1925. The figure shows a distinct increase in the stillbirth rate during the most severe influenza months. More specifically, although the stillbirth rate typically hovered between 20 and 25 stillborn children per 1,000 live-born babies, it increased in October 1918 to a rate of almost 35—an increase very similar to that in the United States (Almond 2006). This increase indicates a certain degree of culling due to the pandemic among fetuses exposed during the second and third trimester. Although the increase was considerable in relative terms, it nevertheless resulted in only a very modest increase of one stillborn child per 100 live births. This conclusion is confirmed by monthly data on live births, showing no indications that the increasing number of stillbirths influenced the size of the affected birth cohort (Fig. A6, online appendix).

Another indicator of potential fetal health selection is the sex ratio at birth, primarily deriving from male fetuses being weaker than female fetuses. If the percentage of male births deviates negatively from the normal 52 % of all live births, it is an indication of a high incidence of spontaneous abortions, which are likely to have been especially common during the first trimester of pregnancy. As the influenza peaked during the last quarter of 1918, if early pregnancy spontaneous abortions surged because of the influenza pandemic, the sex ratio could be expected to be affected from mid-1919 to September/October the same year. Fig. A8 (online appendix) shows the sex ratios of each monthly birth cohort born between January 1916 and December 1920, indicating that the percentage of males consistently hovered at around 51.5 % and never went below 50 % or above 53 % at any time during the period. Furthermore, the shaded area illustrates the sex ratio for the birth cohorts who were in their first trimester of pregnancy during October–December 1918 and consequently the group that should have been the most strongly affected if fetal exposure to the 1918 influenza pandemic caused a change in the sex ratio. The figure shows no indication of an increase in spontaneous abortions and miscarriages as a result of the influenza pandemic.

**Fig. 2:**
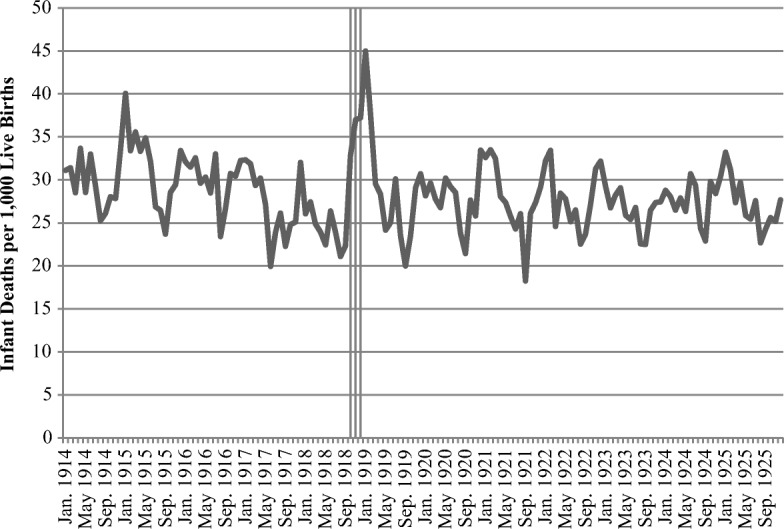
Infant deaths due to malformations and disease, expressed as a share per 1,000 live births during current month, 1914–1925. The shaded vertical bars represents the period October–December 1918. *Source:* Sveriges Officiella Statistik: Befolkningsrörelsen 1914–1925 and Dodsorsaker 1914–1925.

Our last indicator of fetal selection is monthly data on infant deaths due to malformations and disease, most of which took place in the first month of life. Because the seasonality of births is strong, the number of deaths due to malformations and disease in a month is expressed in relation to the number of children born in each month. Figure 2 indicates a distinct increase in infant deaths due to malformations and disease during December 1918 to February 1919, peaking in January 1919. More specifically, 45 infants per 1,000 live births in each of these months died from such causes, compared with between 25 and 30 during any normal month. Although we are unable to distinguish between infant deaths directly due to a certain disease (such as the Spanish flu) and deaths due to malformation, we believe that the timing of deaths suggests a dominance of the latter. Had the peak in infant deaths occurred during the peak influenza months, it would have been consistent with the notion that postnatal exposure to the flu resulted in observed infant mortality. Instead, the delayed response suggests an elevated mortality through malformation among infants exposed to the pandemic during the third trimester. Although this increase indeed is dramatic in relative terms, in absolute terms, it translates to about 100 more deaths compared with the average January. Thus, considering that the total number of infant deaths during any given year during this period amounts to about 8,000 and that the increase is only 1.5–2.0 percentage points, survival selection due to the pandemic should still be modest.

Health selection of individuals exposed to the Spanish influenza *in utero* may also become manifest during childhood and early adolescence (and beyond). Based on individual-level data on all deaths in Sweden occurring during the twentieth century, panels a–d of Fig. A9 (online appendix) show cohort age-specific mortality estimates until age 15. These graphs suggest no lingering elevated mortality among individuals born during the first three quarters of 1919, which are the primary groups exposed to the influenza while *in utero*.^3^ In fact, the cohorts exposed to the Spanish flu while *in utero* do not exhibit discernably higher mortality at least until age 15. Although the stillbirth rate and rate of infant mortality due to malformations and disease suggest a certain sensitivity among fetuses exposed during late gestation, there appears to be no indication of persisting health consequences until age 15 years for those who survived past the fetal stage.

The preceding discussion concerns cohorts exposed to the influenza while *in utero* or very early in life, but another potential problem for the analyses concerns birth cohorts 1920–1924, which are used as comparison groups. These cohorts were possibly affected by nontrivial selection mechanisms due to the relaxation of barriers to emigration after WWI, which caused a surge in out-migration from Sweden that peaked in 1923, at approximately 30,000 emigrants. This number should be compared with a low of 5,000 emigrants in 1918 and an outflow of approximately 10,000 annually in 1919–1929 (Befolkningsrörelsen 1911–1929). A similar potential problem applies to the composition of parents choosing to conceive during the years 1920–1923; these parents were potentially affected by the sharp economic downturn affecting Sweden during the early 1920s (Karlsson et al. 2014). As an illustration, the number of job applications recorded by the public authorities during October 1921 amounted to almost 270 % of the number recorded during October 1919. From 1923 and onward, the number of job applications returned to normal levels (Sociala meddelanden 1919–1924), indicating that the birth cohorts conceived from then onward (albeit possibly with a certain delay) should not have been characterized by selection linked to the harsh labor market conditions.

To summarize, the 1918 pandemic was a very deadly and evidently very contagious disease that spread worldwide in a short period. Mortality statistics suggest that the flu affected mainly people in active ages. As far as fetal health and socioeconomic selection is concerned, however, its effect on children appears to have been negligible according to a range of indicators. In terms of health selection, an evident source for concern is the diminished size of the birth cohort conceived during the influenza pandemic. If this had been due to increasing fetal death, it would suggest fetal health selection. The sex ratio at birth, however, remained stable, as did the birth weight, suggesting that those born were similar to surrounding cohorts. Furthermore, stillbirths increased by only 1–1.5 percentage points and would therefore contribute only at the very margin to the diminished size of concerned birth cohorts. The same also applies to infant deaths due to malformation and disease. Overall survival selection effects are therefore expected to be small, which is also shown by the fact that survival to age 15 years was the same for the cohorts born shortly after the flu as for surrounding cohorts.

Another explanation for the smaller birth cohort conceived during the pandemic, and one that is also of great concern, is a shift in the socioeconomic composition of the parents. Indeed, if those conceived during the pandemic differed from the surrounding cohorts’ socioeconomic origin, this would violate the ability to treat the study design as quasi-experimental. The socioeconomic background of the cohorts born during 1918 and 1919 appears to have shifted slightly: the proportion of children born to well-off parents was about 1 percentage point higher than for surrounding cohorts. This shift, however, was very marginal, and it persisted over a longer period than the pandemic, which makes it easier to separate their effects.

In conclusion, although the cohort born nine months after the pandemic was smaller than usual, evidence does not suggest that health or socioeconomic factors are important underlying mechanisms. Instead, because of the strong compensatory fertility response occurring soon after the pandemic, the evidence suggests that the fertility decline was pervasive across all social groups and not the result of factors that would carry with them severe consequences for the validity of the analysis that follows.

## Data and Methods

The data used in the analyses come from the Swedish Interdisciplinary Panel (SIP), administered at the Centre for Economic Demography at Lund University in Sweden and covering all individuals living at any time in Sweden from 1968 until 2012. This study focuses on the population born in Sweden, for whom the SIP contains detailed individual-level information on county, year, and month of birth, as well as information on income and occupational attainment, hospitalization, and cause of death—all of which we can observe after 1968.

The models estimated follow a common logic throughout, with one difference depending on whether the individual is observed once (Eq. (1), for income, occupation, mortality) or repeatedly (Eq. (2), for hospitalization).

1 $$ {y}_i=\upalpha +{\sum}_{j=1}^{3/17}{\upbeta}_j{Exposure}_c^j+{\sum}_{c=1}^{23}{\upgamma}_c County\ {of\ birth}_i^c+{\sum}_{m=1}^{11}{\updelta}_m Month\ {of\ birth}_i^m+{\sum}_{y=1}^{11}{\updelta}_y Year\ {of\ birth}_i^y+{\sum}_{c=1}^{23}{\uptheta}_{1c} County\ {of\ birth}_i^c\times {\sum}_{y=1}^{11}{\updelta}_y Year\ {of\ birth}_i^y+{\upvarepsilon}_i. $$

2 $$ {y}_{it}=\upalpha +{\sum}_{j=1}^{3/17}{\upbeta}_j{Exposure}_c^j+{\sum}_{c=1}^{23}{\upgamma}_c County\ {of\ birth}_i^c+{\sum}_{m=1}^{11}{\updelta}_m Month\ {of\ birth}_i^m+{\sum}_{y=1}^{11}{\updelta}_y Year\ {of\ birth}_i^y+{\sum}_{c=1}^{23}{\uptheta}_{1c} County\ {of\ birth}_i^c\times {\sum}_{y=1}^{11}{\updelta}_y Year\ {of\ birth}_i^y+{\uprho}_1{Age}_{it}+{\uprho}_2 Age\ {squared}_{it}+{\upvarepsilon}_{it}. $$

The key variables in the models are represented by a set of dummy variables (β) for exposure to the influenza pandemic. Depending on the model specification, this is operationalized either only through fetal exposure—contrasting exposure during all three trimesters with exposure for all other birth cohorts—or through fully modeling prenatal and postnatal exposure using a comprehensive set of 17 dummy variables. The former specification is more comparable with that used in the majority of previous studies, albeit more sensitive to model misspecification; the second specification is fully flexible and thus arguably better addresses whether the common trend assumption is reasonable. In addition, because of concerns regarding other exposure ages being selected on unobservable characteristics (e.g., birth cohorts 1918–1919 being selected on socioeconomic background), this second specification allows for the investigation into whether the *in utero*–exposed groups differ from cohorts exposed during different but also potentially important periods or only from a common trend represented by all birth cohorts not exposed while *in utero*.

All model specifications control for county-, month-, and year-of-birth fixed effects, allowing for the year-of-birth effects to vary across counties of birth and with standard errors clustered at the county level. Last, the models exploiting repeated observations on the examined individuals additionally control for age and age squared.

The sample selected for the analysis consists of all individuals born in Sweden between 1914 and 1925. In defining influenza exposure, we exploit both temporal and geographical variation of the spread of the influenza pandemic. This is made possible through the use of county-level mortality data, which allows us to define the timing and duration of the most severe 1918 autumn/winter wave of the pandemic across counties. For each of the 24 counties, we define the onset of the pandemic to occur from the month when the crude death rates (CDR) exceeded 1.75 deaths per 1,000 inhabitants. Although the threshold may appear arbitrary, we would argue that it is not. During 1917 and the first half of 1918, the median CDR was 1.09 and never exceeded 1.74. Using our definition, the onset for all counties occurred between September and November of 1918, with the large majority occurring in October. As expected, and similar to the rest of the world, the difference in the timing of the outbreak between the different counties is limited. The end of the influenza period is defined as occurring from the month when the CDR fell below the threshold of 1.75 deaths per 1,000, which happened no later than February 1919. Fig. A9 (online appendix) displays influenza exposure periods across the Swedish and between-county differences in influenza intensity. The latter is calculated separately by county and is determined based on the highest observed CDR during the pandemic period compared with 12 months before in order to eliminate effects of population age structure. This is important given that the proportion of young adults, who are the most sensitive to influenza, varied between counties. Maximum CDR *increases* in these counties during the influenza months range from 249 % (Halland) to 507 % (Gävleborg). *Low-intensity areas* are defined as counties where the CDR increase does not exceed 300 % (*n* = 9), and *high-intensity counties* are those where the increase in CDR exceeds 400 % (*n* = 6).

Based on the individual’s county and month of birth, we are able to pinpoint when an individual was exposed to the 1918 influenza pandemic with a high degree of accuracy. This allows us not only to identify individuals who were exposed while *in utero* but also to distinguish between trimesters of exposure. This distinction should prove to be important because the fetal growth process is characterized by different stages, implying that the consequences of experiencing an insult may differ depending on the timing of exposure. Given that the duration of the influenza pandemic consistently exceeded one month, an individual can thus have been exposed during more than one trimester of pregnancy. Because we do not have data on the individual’s precise date of birth, we assume that an individual was born on the 15th day of the month and year of birth. Thus, the third, second, and first trimesters of pregnancy for an individual born in March 1919 began (respectively) 90, 180, and 270 days prior: on December 15, September 16, and June 18, 1918, respectively. Thus, as an example, if the influenza pandemic lasted from October to December 1918, the individual in question is considered to have been exposed during the second and third trimesters.

Because the long-term effects of being subjected to the Spanish flu may extend beyond those exposed during the fetal stage, full model specifications explicitly account for direct exposure subsequent to birth and indirect exposure through the mother, occurring prior to conception. Among individuals exposed subsequent to birth, we distinguish between those subjected to the pandemic in six-month intervals at ages 0–24 months. Thereafter and until being exposed at age 5 (60 months), exposure groups are operationalized in one-year intervals. A similar strategy is employed at the opposite end of the range of birth cohorts examined—that is, for individuals who were not themselves directly exposed but who were conceived subsequent to the pandemic. Again, for individuals conceived up to 24 months after the end of the pandemic, exposure is operationalized in six-month intervals; thereafter and until six years after the pandemic (72 months), exposure is measured in one-year intervals.

The dependent variables for the study are represented by a number of socioeconomic as well as health outcomes. Socioeconomic outcomes are examined for males only through income and occupational attainment in 1970, when individuals in the sample were aged 45–56. Information on occupation is provided by the 1970 census and is operationalized through the Cambridge Social Interaction and Stratification (CAMSIS) scale (Prandy 1999). Theoretically, the classification rests on the argument that individuals sharing social positions are more likely to interact with individuals belonging to groups of similar social standing. Although CAMSIS in its truest sense is an ordered interval variable, ranging from 1 to 99, its variability within this range means that it typically is treated as a continuous variable. In the multivariate analysis, ordinary least squares (OLS) is used to examine the extent to which males’ occupational attainment is affected by exposure to the Spanish flu. Income is obtained from the income and tax register and is analyzed by means of Tobit regression to account for the considerable share of censored zero values. The SES outcomes are not operationalized identically, but their comparability should be facilitated by the facts that they were observed at the same point in time and that they are both continuous variables.

Adulthood health is measured among both men and women. When analyzing health outcomes, we use data from 1968 and onward, following individuals from age 54 until age 87, death, or emigration—whichever occurs first. Health is measured through both morbidity and mortality. Morbidity is obtained through annual information on both the duration and the underlying diagnosis of all hospitalization spells. In the analysis, we however rely only on (1) whether individual *i* was hospitalized during year *t*, and (2) the combined duration of all hospitalization spells of individual *i* during year *t*. We do not exploit information on the underlying diagnosis because of left-censoring: we are unable to measure the *onset* of disease accurately given the unavailability of information prior to 1968. A further caveat is that the hospitalization register covers all Swedish counties only from 1987, with coverage gradually increasing from its start in 1968. Thus, individuals are included in the analysis only provided that their county of residence is covered by the hospitalization register during the actual observation year. The analysis examines several outcomes, including the probability of hospitalization during the current year, using binary logistic regression, multinomial logistic regression models distinguishing between different durations of hospitalizations, and Tobit regression models analyzing the number of days of hospitalization.

We perform the mortality analysis using information provided by the death register, covering all deaths from January 1, 1968, until the end of 2012. All individuals are considered at risk from turning 54 years of age and are censored when turning 87 years of age or exiting the data set through emigration. The analysis focuses both on all-cause as well as cause-specific mortality, with principal cause of death categorized as cancer (ICD10: B21, C00-D48, ICD9: 140-239), cardiovascular disease (ICD10: I00-I99, ICD9: 390-459), respiratory (ICD10: J00-J99, ICD9: 460-519), or other diseases (remaining ICD9/10 codes).

The ability to follow individuals from as early as 1968 is one factor distinguishing this study from ones for the United States (Almond and Mazumder 2005; Mazumder et al. 2010; Myrskylä et al. 2013), whose observation windows begin in 1982 or later. This has potentially important implications because the relative importance of a given cause of death is not constant across the life course. Data on the entire population in SIP indicate that for Sweden and the period 1968 onward, among the four cause-of-death categories examined here, cancer mortality was responsible for the largest share of all deaths in the age groups 50–59 and 60–69; beyond those ages, the dominant cause of death was cardiovascular disease. Consequently, this study differs from the aforementioned studies on the United States by examining individuals from an earlier age, before cardiovascular mortality has become the dominant cause of death. We estimate the models for both the overall and the cause-specific mortality using Gompertz survival analysis.

## Descriptive Statistics

The analytical samples differ somewhat depending on the outcome variable being examined (see Table A1, online appendix). Common across all samples are the restriction of the population to individuals born in 1914–1925 and the definition of influenza exposure. Across all samples, the population is centered at 1919, and each annual birth cohort represents a roughly similarly sized share of the analytical sample. In addition, the share of individuals exposed to the 1918 influenza pandemic while *in utero* is highly similar across the samples analyzed (Table A1): approximately 4 % of each study sample was exposed during one of the trimesters of gestation.

The analyses of socioeconomic outcomes—income and occupational attainment—are cross-sectional and are observed for males in 1970. Table A1 shows that the mean income, observed for ages 45–56, amounts to 34,900 SEK (in 1970 prices) and varies considerably (SD = 28,100 SEK). The mean CAMSIS score of 47.4 (SD = 14.6) indicates that the sample well represents the typical population for which the classification was derived: the default CAMSIS classification was designed to have a mean of 50 and a standard deviation of 15 in a nationally representative population. Of greater interest is, however, the extent to which the data indicate that exposure to the influenza pandemic is associated with later-life adversity.

**Fig. 3:**
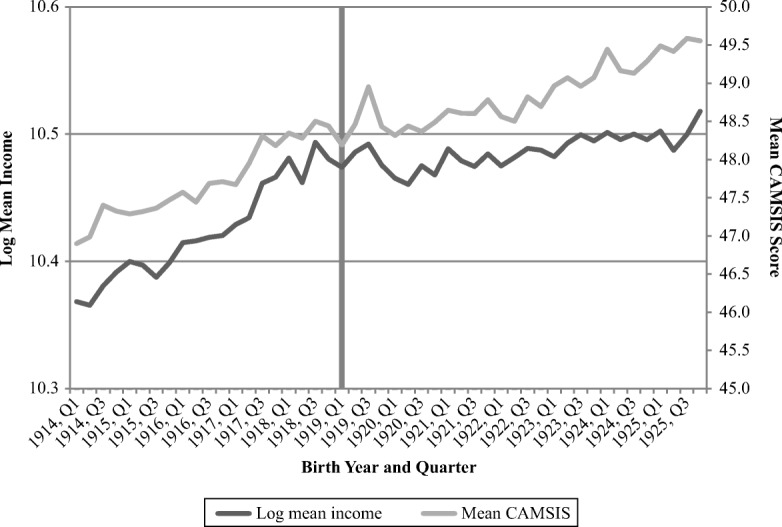
Log mean income and mean CAMSIS score in 1970, by birth year and quarter. CAMSIS ranges from 1 to 99, with higher scores indicating higher occupational attainment. The shaded vertical bar represents individuals born during Quarter 1, 1919. *Source:* Swedish Interdisciplinary Panel.

Figure 3 displays the log mean attained income (left axis) as well as the mean CAMSIS score (right axis) in 1970, for all year and quarters of birth examined herein. The dark gray vertical line represents the cohort born during the first quarter of 1919; that cohort along with cohorts born during the second quarter and (albeit to a lesser extent) the third quarter that same year were the cohorts principally exposed to the pandemic while *in utero*. For inflation-adjusted log mean income, the data reveal a more rapid increase before 1917 than after 1920. More interestingly, the data show an upswing from the end of 1917 to 1919, just like the socioeconomic composition of fathers, with a small drop for children born in the first quarter of 1919. Similar drops appear, however, both before and afterward the first quarter of 1919, indicating that this quarter’s cohort fails to stand out in any particular way. Thus, the income pattern is quite different from what was found for the United States, where the cohort exposed to the influenza pandemic while *in utero* was characterized by a pronounced decline (Almond 2006), at least partly because of a change in the social gradient of parents (Brown and Thomas 2013). A virtually identical pattern can be observed for occupational status attainment.

Table A1 shows that men experienced greater morbidity across all measurements of hospitalization, as would be expected. Although the differences between the sexes are not dramatic, in the age range examined for all cohorts (54–87), about 19 % and 17 % of men and women, respectively, experienced a hospitalization spell during any given year.

**Fig. 4:**
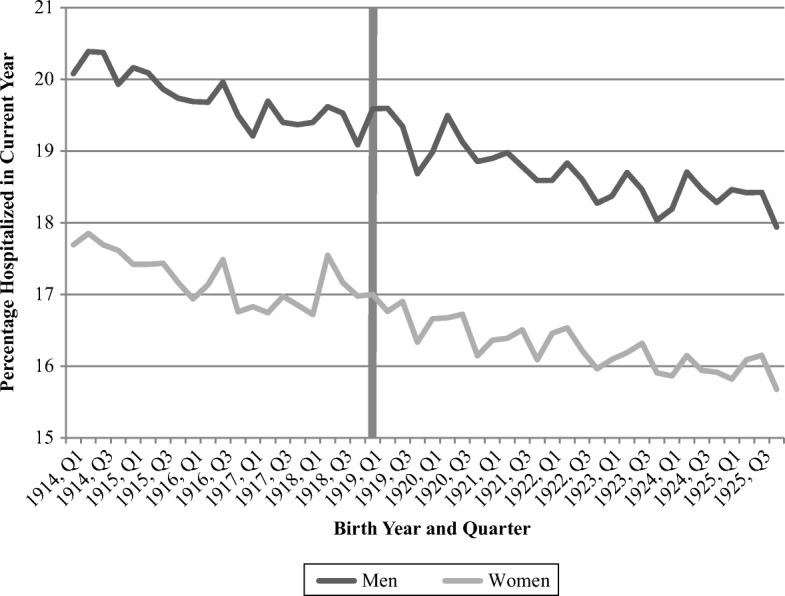
Share hospitalized during current year, by birth year and quarter. The shaded vertical bar represents individuals born during Quarter 1, 1919. *Source:* Swedish Interdisciplinary Panel.

Turning to whether exposure to the Spanish flu pandemic is linked to later-life adversity, Fig. 4 displays a generally declining probability of hospitalization across birth cohorts for both men and women. We find no indications of increased risk of hospitalization resulting from fetal exposure to the influenza pandemic for either sex. Using another metric, the mean days of hospitalization (Fig. A11, online appendix) renders a similarly monotonically decreasing trend, from 3.8/4.0 days for the female/male 1914 first quarter cohort to 2.3/2.5 for the 1925 fourth quarter cohorts. Thus, neither shows any indication that the cohorts born during the first quarters of 1919 experienced any different hospitalization outcomes than surrounding cohorts.

The last two columns of Table A1 display the summary statistics for the mortality analysis for the sample of individuals observed at age 54 and followed until death, age 87, or censoring through emigration (whichever occurs first). Overall, males have higher mortality: 75 % for males and 57 % for females. The data also indicate that cardiovascular disease is the most common primary cause of death, representing more than double the share of deaths due to cancer. The share of deaths due to respiratory disease for both sexes is roughly as large as that of other causes of death, at about 5 % of all deaths.

**Fig. 5:**
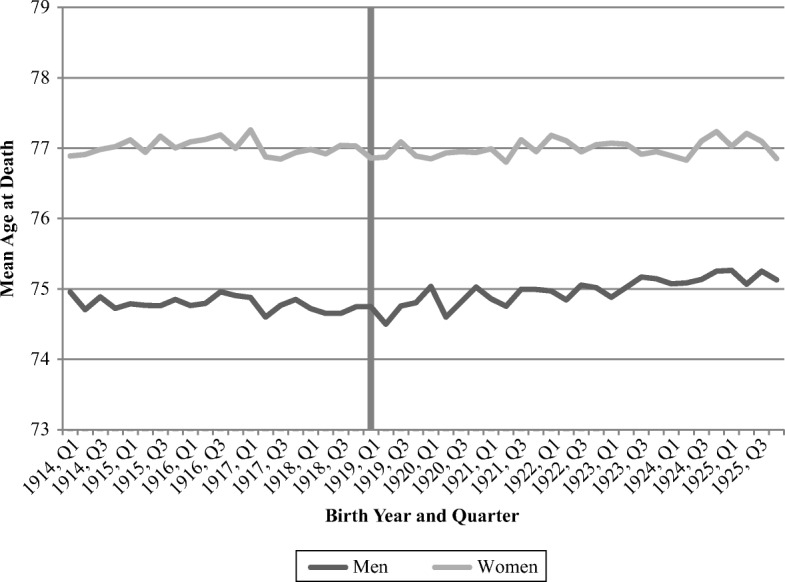
Mean age at death, by birth year and quarter. The shaded vertical bar represents individuals born during Quarter 1, 1919. *Source:* Swedish Interdisciplinary Panel.

Figure 5 shows the development of the mean age at death for the examined birth cohorts, conditional on survival until 1968, again displayed by quarter of birth. Although the trend shows an increasing mean age at death over time, it is indeed quite modest and not indicative of any noteworthy deviation from the overall trend experienced by the cohort born during the first quarter of 1919.

## Results

### Income Attainment

Table 1 presents the results from Tobit regression analyses on males’ income attainment in 1970. Model 1 is limited to the consequences of influenza exposure during the three trimesters of pregnancy, investigating the extent to which exposure during one or more of these periods resulted in experiencing a significant deviation from the overall trend as represented by all other birth cohorts 1914–1925, who were not exposed during the fetal stage. The results fail to provide any consistent evidence suggesting that *in utero* exposure to the 1918 influenza pandemic negatively affected individuals’ income attainment. Whereas the point estimate for late pregnancy exposure (the third trimester) is negative, it is far from statistically significant at conventional levels. Point estimates for exposure during remaining periods are positive and are also statistically insignificant, with 95 % confidence intervals that overlap both each other and the negative point estimate for exposure in the third trimester.

**Table 1:** Results for income and occupational attainment: Tobit regression results with income in 1970 (in 1,000 SEK) as the dependent variable (Models 1 and 2) and OLS regression results with CAMSIS score in 1970 as the dependent variable (Models 3 and 4)

Exposure Period	Model 1	Model 2	Model 3	Model 4
49–60 Months After Birth		–0.188		–0.0811
37–48 Months After Birth		0.209		–0.158
25–36 Months After Birth		–0.275		–0.0387
19–24 Months After Birth		–0.149		0.108
13–18 Months After Birth		0.593*		0.237
7–12 Months After Birth		0.0263		0.352**
0–6 Months After Birth		0.686		0.404*
Third Trimester	–0.293	–0.471^†^	0.0288	0.118
Second Trimester	0.457	0.346	0.0544	0.0933
First Trimester	0.451	0.518^†^	0.451*	0.616**
0–6 Months Before Conception		–0.104		0.0448
7–12 Months Before Conception		0.351		–0.0305
13–18 Months Before Conception		0.147		–0.0955
19–24 Months Before Conception		–0.186		0.0569
25–36 Months Before Conception		–0.190		–0.169^†^
37–48 Months Before Conception		–0.00422		–0.125
49–60 Months Before Conception		0.107		–0.0194
61–72 Months Before Conception (ref.)				
Number of Observations	450,245	450,245	420,985	420,985

*Note:* The CAMSIS score ranges from 1 to 99, with higher scores indicating higher occupational attainment.

^†^*p* < .10; *p < .05; **p < .01

The empirical strategy in Model 1 relies on an assumption that cohorts not exposed to the influenza pandemic while *in utero* can be appropriately modeled through a deviation from joint effects of county and year of birth. This is not necessarily ideal because of, for example, the changing socioeconomic composition of fathers across birth cohorts. To more fully exploit the information at our disposal, Model 2 applies a fully flexible specification of pre- and postnatal exposure to the Spanish flu, being otherwise analogous to Model 1. The reference category is individuals who were conceived 60–72 months after the influenza pandemic. This period occurred subsequent to the economic crisis and emigration upsurge affecting the 1920–1924 birth cohorts, as discussed previously. The model indicates substantial fluctuations across birth cohorts, also substantively confirming that the results obtained for Model 1—namely, that individuals exposed during the first and second trimesters enjoyed an earnings *advantage*—were not due to the specification of Model 1. More specifically, compared with individuals conceived 60–72 months after the end of the pandemic, those exposed during the first trimester of pregnancy enjoyed a statistically significant 518 SEK higher income, translating to roughly 1.5 % of the mean, which is not inconsistent with the elevated share of high-SES fathers for these cohorts. Indeed, a more relevant comparison given the individuals’ changing socioeconomic backgrounds is between individuals exposed while *in utero* and those born during early 1918, who are expected to be more similar in terms of their background. The effect for individuals exposed during the third trimester (born during late 1918 or early 1919) is negative and thus in line with expectations that fetal exposure to the Spanish flu caused later-life disadvantage. Effects, however, are not estimated with sufficient precision to be statistically significantly different from the closest surrounding birth cohorts, which are expected to be characterized by similar background characteristics.^4^

### Occupational Status

An outcome representing a different yet related measurement of labor market performance is occupational status attainment, operationalized through CAMSIS. Here, we estimate the effects of influenza exposure using OLS regression. Similar to the analyses of income, the outcome is measured using data from the 1970 census, when individuals in the sample are aged 45–56. As shown in Table 1, the results are consistent with those previously obtained for income attainment in terms of the absence of evidence showing that fetal exposure to the 1918 influenza pandemic is associated with adverse socioeconomic outcomes. Similar to the results for income, the strongest effect observed is among males who were exposed during the first trimester. More specifically, compared with all surrounding exposed birth cohorts, exposure during the first trimester is associated with a 0.45-unit increase in the CAMSIS score according to Model 3. The effect of exposure during the second and third trimesters is statistically insignificant but positive.

Regarding the fully flexible specification of effects of influenza exposure on CAMSIS attainment, results from Model 4 are similar to those of the income attainment analyses in that exposure during infancy is associated with a higher CAMSIS attainment. Overall, however, the size of the effects should not be exaggerated. Indeed, according to Model 4, first trimester exposure to the Spanish flu increases the predicted CAMSIS score only by 0.62, translating to 1.3 % of the mean. Again, the higher occupational status predicted for these cohorts is almost perfectly associated with the increase in the share of well-off parents in 1918–1919 and not to the pandemic itself. Similar to the analyses on income attainment—arguably, even more evidently so—the positive effects are considerably smaller for the cohorts exposed during the second and third trimesters, possibly due to exposure to the Spanish flu. Again, however, because the effects are not estimated with enough precision for us to conclude that exposure during the second or third trimester is significantly different from that of surrounding cohorts’, the results remain inconclusive.

### Hospitalization

Table 2 presents hospitalization results for males, with hospitalization during any given year between the ages of 54 and 87 operationalized in several different ways. With the results regarding socioeconomic outcomes in mind—namely, that males exposed to the Spanish flu while *in utero* or in the first 18 months of life experienced significantly higher incomes and higher CAMSIS occupational attainment—the results for males on morbidity through hospitalization are interesting. Model 1 displays odds ratios for the probability of hospitalization during the current year estimated using binary logistic regression. Following the same logic as before, the model estimates how *in utero*–exposed cohorts deviate from common birth county and year effects. This model suggests that males exposed during the second trimester experience a statistically significant 3.5 % increase in the odds of hospitalization. Model 3 is otherwise analogous but uses Tobit regression to estimate the effect on the number of days of hospitalization during the current year. Although difficult to directly compare the size of the obtained coefficients, the model again suggests increased morbidity among the males exposed during the second trimester. The coefficient, which is statistically significant at the 1 % level, shows a predicted increase of 0.81 days of hospitalization during any given year. Despite the modest magnitude of the effect in absolute terms, this translates to a nontrivial 25 % increase at the mean.

**Table 2:** Results for hospitalization among males: Odds ratios from logit regression models with hospitalization during the current year as the dependent variable (Models 1 and 2); Tobit regression results with days of hospitalization during the current year as the dependent variable (Models 3 and 4); and relative risk ratios from multinomial logit regression with days of hospitalization (categories) during the current year as the dependent variable (Models 5 and 6)

					Model 5					Model 6				
Exposure Period	Model 1	Model 2	Model 3	Model 4	1–5 Days	6–10 Days	11–20 Days	21–30 Days	>30 Days	1–5 Days	6–10 Days	11–20 Days	21–30 Days	>30 Days
49–60 Months After Birth		1.021		0.696						0.975	1.019	1.001	1.103**	1.105**
37–48 Months After Birth		1.008		0.248						0.995	1.004	1.018	1.046^†^	1.013
25–36 Months After Birth		1.020		0.324						1.009	1.026	1.031	1.059^†^	1.010
19–24 Months After Birth		1.010		0.273						0.998	0.999	1.032*	1.006	1.028
13–18 Months After Birth		1.018		0.481^†^						1.006	1.002	1.045**	1.002	1.047*
7–12 Months After Birth		1.012		0.216						1.012	1.026^†^	1.014	1.017	0.991
0–6 Months After Birth		1.000		0.120						0.994	1.000	0.997	1.018	1.016
Third Trimester	1.003	1.009	0.0206	0.137	1.008	0.988	1.006	1.017	0.999	1.015	1.002	1.016	1.025	0.993
Second Trimester	1.035**	1.029^†^	0.806**	0.778*	1.022	1.041*	1.032	1.017	1.078**	1.014	1.036	1.021	1.009	1.085*
First Trimester	0.996	0.995	–0.214	–0.231	1.018	0.993	0.989	0.954*	0.973	1.017	0.995	0.989	0.951*	0.971
0–6 Months Before Conception		0.997		0.0324						0.993	1.004	0.993	0.998	1.003
7–12 Months Before Conception		1.012		0.283						1.001	1.007	1.021	1.000	1.045
13–18 Months Before Conception		1.002		0.132						0.994	1.010	0.993	1.013	1.019
19–24 Months Before Conception		0.985		–0.265						0.986	0.984	0.971	0.990	1.000
25–36 Months Before Conception		0.994		–0.128						0.986	1.003	0.991	1.010	1.003
37–48 Months Before Conception		0.985^†^		–0.460**						0.986	1.009	0.983	0.954*	0.966^†^
49–60 Months Before Conception		0.977**		–0.466*						0.973**	0.986	0.975	0.972	0.981
61–72 Months Before Conception (ref.)														
Number of Observations	9,077,746	9,077,746	9,077,746	9,077,746	9,077,746					9,077,746				

^†^*p* < .10; *p < .05; **p < .01

We also estimate models that fully exhaust the different possible periods of exposure before and after birth. Comparing Model 2 with Model 1 provides an estimate of the probability of any hospitalization during the year. The results again suggest similar point estimates for *in utero*–exposed cohorts. Exposure during the second trimester resulted in a 2.9 % increase in the odds of hospitalization, which only very slightly attenuated compared with Model 1. The Tobit analysis on the number of days of hospitalization in Model 4 yields a similar conclusion. Exposure to the Spanish flu during the second trimester of pregnancy is linked to a statistically significant increase of 0.78 days of hospitalization during any given year. Arguably of equal importance is that although SES attainment was elevated also for those born during the 18-month period prior to the pandemic, the health of only those affected during the second trimester was affected. Also noteworthy is that this deviation is larger than for any other birth cohort. Being exposed to the flu during the second trimester thus stands out whether the comparison is the trend or individual birth cohorts.

In order to explore potential nonlinearities in the relationship between exposure to the Spanish flu and days of hospitalization, results from multinomial logistic regression models are presented as Models 5 and 6 for males. The results from Model 5 are similar to those already discussed, systematically suggesting that second trimester exposure is linked to increased morbidity. More specifically, point estimates for shorter spells indicate roughly a 2 % to 4 % increase in the relative risk ratio, although it is not estimated with enough precision to be statistically significant. This is to be contrasted to the 8 % increase in the relative risk ratio of experiencing a hospitalization spell lasting more than 30 days, suggesting that the hospitalization results are driven by comparatively more serious conditions. Finally, the results of Model 6 for the multinomial logit regression are somewhat less clear, possibly linked to the comparatively more complex and noisier nature of the data as a result of estimating the effects across five unique values on the outcome variable.

The results for women, displayed in Table 3, are less consistent than those obtained for males. The results for women do not consistently suggest one common key period of uterine exposure to the influenza pandemic that is associated with adverse later-life health, although the majority of the statistically significant effects are for exposure during the third trimester. Model 1 shows the odds ratios for any hospitalization during the year, indeed suggesting that females who were exposed during the last trimester of pregnancy constitute the group that was disadvantaged; these women had a 2.5 % increase in the odds of hospitalization, with remaining periods of *in utero* exposure rendering null results. Model 2 extends the model to a fully flexible specification, resulting in a similarly sized coefficient but one estimated with less precision.

**Table 3:** Results for hospitalization for females: Odds ratios from logit regression models with hospitalization during the current year as the dependent variable (Models 1 and 2); Tobit regression results with days of hospitalization during the current year as the dependent variable (Models 3 and 4); and relative risk ratios from multinomial logit regression with days of hospitalization (categories) during the current year as the dependent variable (Models 5 and 6)

					Model 5					Model 6				
Exposure Period	Model 1	Model 2	Model 3	Model 4	1–5 Days	6–10 Days	11–20 Days	21–30 Days	>30 Days	1–5 Days	6–10 Days	11–20 Days	21–30 Days	>30 Days
49–60 Months After Birth		0.994		–0.119						0.983	1.019	1.009	0.955	0.984
37–48 Months After Birth		0.984		–0.394						0.975^†^	1.004	0.982	0.968	0.991
25–36 Months After Birth		1.006		0.209						1.010	0.996	1.014	0.970	1.024
19–24 Months After Birth		0.982^†^		–0.426^†^						0.993	0.973*	0.975	0.987	0.980
13–18 Months After Birth		0.994		–0.00928						0.980^†^	1.003	0.978	1.025	1.016
7–12 Months After Birth		1.007		0.150						1.008	1.016	1.004	0.987	1.010
0–6 Months After Birth		1.013		0.328^†^						1.023^†^	1.009	1.004	0.999	1.017
Third Trimester	1.025*	1.024^†^	0.357^†^	0.329	1.033^†^	1.025	1.032*	1.028	1.000	1.027	1.031^†^	1.031^†^	1.024	1.003
Second Trimester	1.003	1.007	0.236	0.371	0.995	1.014	0.988	1.014	1.016	0.989	1.020	0.991	1.049	1.033
First Trimester	1.017	1.018	0.252	0.290	1.015	1.037^†^	1.006	1.029	0.999	1.017	1.041*	1.006	1.030	1.000
0–6 Months Before Conception		1.003		0.116						0.989	1.011	0.998	1.033	1.019
7–12 Months Before Conception		1.013		0.336						0.994	1.025	1.018	1.033	1.025
13–18 Months Before Conception		0.996		–0.0671						0.981^†^	1.018	0.991	1.018	0.998
19–24 Months Before Conception		1.011		0.247						1.016	0.997	1.000	1.046^†^	1.015
25–36 Months Before Conception		0.999		–0.166						0.996	1.020^†^	0.994	1.016	0.970
37–48 Months Before Conception		0.984		–0.328						0.986	0.995	0.970*	0.976	0.986
49–60 Months Before Conception		0.990		–0.167						0.982*	1.008	0.988	0.997	0.986
61–72 Months Before Conception (ref.)														
Number of Observations	11,281,861	11,281,861	11,281,861	11,281,861	11,281,861					11,281,861				

^†^*p* < .10; *p < .05

Models 3 and 4 largely indicate a rather similar story, with females exposed to the Spanish flu during the third trimester experiencing a statistically significant 0.36 more days of hospitalization during any given year, according to the more restricted Model 3. When the model is extended to account for all exposure periods with full flexibility (Model 4), the effect not only diminishes in size (0.33) but also becomes statistically insignificant. Last, the results from Models 5 and 6 suggest a considerably more inconsistent pattern compared with that obtained for males. More specifically, significant and positive effects are found not only for third trimester exposure but also for first trimester exposure as well as pre-conception and postnatal exposure. Interestingly, and in contrast to the results for males, we find no significant results for females for *in utero* exposure beyond 11–20 days.

### Mortality

The mortality analyses focus on both all-cause and cause-specific mortality. As in the morbidity analyses, individuals are considered at risk when they turn age 54 and are subsequently followed until death, age 87, or censoring through emigration—whichever occurs first. The modeling strategy follows that employed in previous sections: Models 1 and 3 in Table 4 estimate the change in the mortality hazard among men and women who were exposed *in utero*, with all remaining birth cohorts representing the reference category. Model 1 for men again indicates that exposure during the second trimester is the period linked to poorer health through a statistically significantly elevated mortality hazard of 3.1 %. Indeed, exposure during remaining trimesters indicates a slight reduction in the mortality hazard, which is statistically significant for the first trimester. The corresponding Model 3 for women fails to indicate any statistically significant results; all hazard rates are close to 1.

**Table 4:** Hazard rates from Gompertz survival analysis models, with all-cause mortality during the current year as the dependent variable

Exposure Period	Model 1 Men	Model 2 Men	Model 3 Women	Model 4 Women
49–60 Months After Birth		0.980		0.997
37–48 Months After Birth		1.011		0.998
25–36 Months After Birth		1.004		1.033*
19–24 Months After Birth		1.005		1.005
13–18 Months After Birth		1.000		1.027^†^
7–12 Months After Birth		0.999		1.015
0–6 Months After Birth		1.009		1.004
Third Trimester	0.986	0.983	1.014	1.024
Second Trimester	1.031*	1.021	1.003	1.004
First Trimester	0.974*	0.973*	0.999	1.000
0–6 Months Before Conception		0.987		1.012
7–12 Months Before Conception		1.025		0.998
13–18 Months Before Conception		1.003		1.006
19–24 Months Before Conception		0.959*		0.983
25–36 Months Before Conception		0.992		0.989
37–48 Months Before Conception		0.982		1.005
49–60 Months Before Conception		0.980		1.014
61–72 Months Before Conception (ref.)				
Number of Observations	442,521	442,521	471,547	471,547

^†^*p* < .10; *p < .05

Proceeding to the fully flexible specification in Models 2 and 4, investigating more carefully whether those exposed *in utero* differ from surrounding cohorts, the results for both sexes remain largely the same. The hazard rate for males exposed during the second trimester is attenuated in terms of both size and statistical significance, thus questioning the conclusion that exposure resulted in an increased all-cause mortality hazard. The results for women in Model 4 again fail to provide any indications that fetal exposure to the Spanish flu resulted in an elevated mortality hazard.

We turn last to cause-specific mortality, presented in Table 5. Models 5–8 focus separately on each examined cause of mortality: cardiovascular disease, cancer, respiratory disease, and other causes. These models are estimated on the male sample, considering only fetal exposure to the 1918 influenza pandemic. The results indicate that the overall mortality increase linked to second trimester exposure is driven by cancer and, to a lesser extent, cardiovascular disease mortality. More specifically, for second trimester exposure, the hazard rate of cancer mortality increases by a quantitatively and statistically significant 9 %; the comparative increase for cardiovascular disease mortality is 3.4 %, which is significant only at the 10 % level. Interestingly, influenza exposure during early pregnancy *lowers* the hazard of mortality by 3.8 % for cardiovascular disease and by a nontrivial 10.5 % for other causes among those exposed during the second trimester. Again, the baseline risk for other causes of mortality needs to be taken into account, implying that the change in absolute terms is rather marginal. For respiratory disease mortality, the regression results suggest null effects. The estimates for females from Models 9–12 again indicate that the link between fetal exposure and later life health is considerably weaker, with point estimates that consistently are statistically insignificant.

**Table 5:** Hazard rates from Gompertz survival analysis models, with cause-specific mortality during current year as the dependent variable

	Men				Women				Men				Women			
Exposure Period	Model 5 Cancer	Model 6 Cardiovascular Disease	Model 7 Respiratory Disease	Model 8 Other	Model 9 Cancer	Model 10 Cardiovascular Disease	Model 11 Respiratory Disease	Model 12 Other	Model 13 Cancer	Model 14 Cardiovascular Disease	Model 15 Respiratory Disease	Model 16 Other	Model 17 Cancer	Model 18 Cardiovascular Disease	Model 19 Respiratory Disease	Model 20 Other
49–60 Months After Birth									0.979	1.017	0.823*	0.910	0.957	1.016	1.048	0.940
37–48 Months After Birth									0.990	1.035^†^	0.957	0.989	0.989	0.997	1.014	1.016
25–36 Months After Birth									0.990	1.004	1.067	0.988	1.046	1.048*	0.911^†^	1.039
19–24 Months After Birth									0.991	1.017	0.994	0.981	1.056*	0.996	0.954	0.963
13–18 Months After Birth									0.965	1.013	1.011	1.033	1.069*	1.019	0.947	1.035
7–12 Months After Birth									0.982	1.006	0.985	1.028	1.007	1.025	1.041	0.964
0–6 Months After Birth									1.025	0.996	1.036	1.019	1.030	1.005	0.967	0.960
Third Trimester	0.966	0.986	1.030	1.019	1.007	1.023	1.027	0.975	0.945*	0.994	1.005	1.025	1.006	1.041^†^	1.044	0.974
Second Trimester	1.088**	1.034^†^	0.957	0.895*	0.993	1.013	0.979	0.996	1.068^†^	1.031	0.920	0.894^†^	0.994	1.013	0.985	1.000
First Trimester	0.983	0.962*	1.011	0.976	0.994	1.004	0.994	0.997	0.984	0.958*	1.017	0.988	0.997	1.004	1.003	0.991
0–6 Months Before Conception									0.966	1.002	0.952	1.000	1.008	1.019	0.993	1.004
7–12 Months Before Conception									1.060^†^	0.994	1.021	1.142*	1.042	0.970	1.010	1.015
13–18 Months Before Conception									0.996	1.000	0.974	1.087*	0.999	1.013	0.994	1.003
19–24 Months Before Conception									0.961	0.950*	0.972	1.002	0.974	0.958^†^	1.138*	1.001
25–36 Months Before Conception									1.000	0.977	1.048	1.009	0.971	0.995	1.021	0.976
37–48 Months Before Conception									0.970	0.978	1.036	0.982	1.011	1.006	0.983	0.997
49–60 Months Before Conception									0.985	0.980	0.959	0.976	1.022	1.023	0.921	1.026
61–72 Months Before Conception																
Number of Observations	442,521	442,521	442,521	442,521	471,547	471,547	471,547	471,547	442,521	442,521	442,521	442,521	471,547	471,547	471,547	471,547

^†^*p* < .10; *p < .05; **p < .01

Finally, we employ the fully flexible specification for influenza exposure. For males, the results from Models 13–16 further reinforce the conclusion that fetal exposure to the Spanish flu is only statistically significantly linked to an increased mortality hazard in cancer and only for exposure during the second trimester. While slightly attenuated, according to Model 13, second trimester exposure statistically significantly increases the hazard of cancer mortality by 6.8 %. The increase in cardiovascular mortality risk for second trimester exposure remains largely similar in size to that in the more basic model specification but becomes statistically insignificant. For females, similar to previous findings on health, results from Models 17–20 show that the largest effects for *in utero* exposure again are for late gestation, in particular for cardiovascular disease and respiratory disease mortality; only the effect of cardiovascular disease mortality is statistically significant.

### Robustness Analyses

Although the results tell a rather consistent story—in particular, for males’ health outcomes—nevertheless, the possibility exists that our main estimates are sensitive to choices pertaining to comparison group and study sample. Indeed, this seems to apply to the results for occupational and income attainment, which is reflected not only in a less clear relationship between *in utero* influenza exposure in terms of our main results but also through point estimates being more volatile to changes in how the outcome variable and study sample are defined. We estimate models on income and occupational attainment using alternative definitions of the outcome variable. For income, we use OLS regression with the natural logarithm of the individual’s income as the outcome variable, and logistic regression with positive income in 1970 as the dependent variable. No trimester of influenza exposure affects later-life income attainment, regardless of specification. For occupational attainment, using CAMSIS recorded in the 1960 census as the outcome yields results that are essentially identical to our main estimates. A logistic regression analysis on high occupational attainment in 1970 suggests that both the first and second trimesters appear to have benefited from pandemic exposure. Turning to mortality, we estimate models identical to those discussed in the main results section but instead using Cox proportional hazards regression or Weibull survival analysis. The results are identical to those of the main analyses: health outcomes are considerably more robustly linked to influenza exposure.

To test the overall robustness of the results, we define the influenza period using alternate thresholds and setting the individual’s date of birth to occur on the first, the last, or a random day of the month. The main analyses consider the influenza pandemic to have started in the month when the county-level crude mortality exceeded 1.75, whereas the robustness analyses apply thresholds of 1.5 and 2.0. Naturally, these different thresholds may prolong or shorten the influenza period for a given county and therefore also influence the population considered as exposed. Given the severity of the influenza pandemic, changing the threshold in most cases does not alter the exposure period and in rare cases alters it only marginally. Similarly, changing the date of birth influences the individual’s period of exposure only at the margin. Furthermore, neither change in definition of exposure to the 1918 influenza pandemic affects the conclusions in terms of economic or statistical significance.

Another threat to our conclusions is that the comparison groups (i.e., individuals not exposed during the fetal stage) were not, as we argue, representative of birth cohorts during what could be considered normal years. Although the fully flexible specification that we rely on for our conclusions indeed fails to indicate that any of the surrounding birth cohorts systematically diverged from the trend, we estimate all models while excluding birth cohorts 1920–1924, who were born during times of high unemployment and an upsurge in emigration. Again, this exercise yields results that are essentially identical to those of the main analyses and therefore does not alter our conclusions.

Compared with several prior studies on the effects of fetal exposure to the Spanish flu pandemic, the effects obtained for fetal exposure are rather modest in size. To explore whether the main effects are disguising differences in the response to pandemic exposure depending on the intensity of influenza exposure, we examine whether the effects differ between low-/medium-/high-mortality counties, as discussed in the Data and Methods section (see Fig. A10, online appendix). Overall, the models fail to indicate that one type of influenza exposure is driving the effects. One possible exception is that the effects of exposure the second trimester on men’s health in the main analysis most consistently appear to be driven by medium-intensity exposure.

## Summary and Discussion

The immediate mortality consequences of the 1918 influenza pandemic were dramatic. In addition, several studies by epidemiologists, demographers, and economists showed negative effects on socioeconomic and health outcomes in later life among individuals exposed during key developmental phases, most notably the fetal stage. Thus, the consequences of the Spanish flu eclipse any subsequent influenza outbreak to date: both the short- and long-term consequences of later influenzas have been comparatively trivial (Fell et al. 2017). Considerable challenges are associated with examining the effects of a single event, such as the 1918 influenza pandemic, that co-occurred with an event as tumultuous as a world war, which is associated with many other factors that might influence later-life health. Thus, it is essential that the assertions behind a quasi-experimental design are fulfilled.

Our study addresses these assertions by using an unprecedented number of sources of contemporary statistics. More specifically, we address these issues using annual or monthly statistics on parental SES, stillbirths, sex ratio at birth, birth weight, and cohort mortality in childhood and early adolescence. We remain confident that neither indicator of selection addressed indicates that the results are driven by such issues to any significant extent, but it is nevertheless important to acknowledge that fully exhausting this possibility is difficult. Another potential caveat is linked to issues of multiple significance—that is, that statistically significant results are obtained purely by chance. The consistency of our findings, however, argues against this as the underlying factor behind the results.

We exploit Swedish longitudinal individual-level data with detailed information on time and place of birth to identify influenza exposure with a high degree of accuracy. Along with data on adulthood income, occupational attainment, hospitalization, and mortality by cause of death, we are able to model not only the timing of influenza exposure but also the outcomes thereof in a comprehensive fashion. To our knowledge, this study is the first to use longitudinal information on hospitalization in a study of the long-term impact of the 1918 pandemic as a complement to mortality by cause of death. Furthermore, our study uses longitudinal data from 1968 onward to cover the entire population of interest, thereby avoiding potential sampling problems.

Our results on health outcomes are consistent with the fetal origins hypothesis: exposure to the 1918 influenza while *in utero* resulted in higher morbidity at ages 54–87, as measured by hospitalization. For men, the results also support a link to elevated mortality. That being said, the results for mortality in particular are rather modest in terms of size and, especially for women, are somewhat noisy. For males, exposure during the second trimester stands out both for hospitalization and mortality. For the latter, we find effects for mortality due to cancer, with substantially weaker effects for all-cause mortality as well as for cardiovascular disease mortality. The most basic model specification finds a 3.1 % higher all-cause mortality risk for those exposed, translating to about three months shorter remaining life expectancy at ages 54–87. Albeit not a trivial health penalty, the size of the effect is only slightly larger than the difference in life expectancy at birth from being born about a year earlier and is hardly expected to influence the individual’s capabilities and well-being to any noteworthy extent. Even when we take into account that not everyone was affected by the flu and that the effects need to be modified accordingly, the effect is moderate, not exceeding five months. This estimate is calculated based on indications from contemporary accounts reported in this article suggesting that as many as 75 % of those at reproductive ages contracted the disease, compared with 20 % to 60 % during the typical seasonal influenza.

For females, we find effects of exposure during the last trimester on the incidence and length of hospitalization but no effect on overall or cause-specific mortality. Thus, although males were more vulnerable to long-term health effects of the 1918 pandemic during the middle part of fetal development, females’ sensitive period occurred during late gestation but with effects that are limited to morbidity outcomes.

Previous research using U.S. data to analyze heart disease and diabetes found that among those aged 60–82, the effects of fetal exposure to the influenza pandemic on later-life heart disease were stronger for males than for females (Mazumder et al. 2010). Another study using U.S. survey data found effects on both coronary heart disease and cancers at ages 75–84 (Myrskylä et al. 2013). Thus, similar to this study, exposure to the Spanish flu during pregnancy has been shown to affect the incidence of not only heart disease but also of cancer later in life, with effects predominantly being found for males. Compared with previous studies, our study finds stronger effects on cancer in particular. A possible explanation for this difference may be that we analyze individuals at younger ages, including ages when cancer is the dominant cause of death. Differences in results may also depend on the context—for example, in terms of lifestyle factors. Smoking, which differs across cohorts and countries, is known to interact with other risk factors for cancer, diabetes, and coronary heart disease (Mills et al. 2011).

Our results consistently show that any health adversity is limited to males and females exposed to the influenza pandemic during mid- or late pregnancy, respectively, and to the first months on life for females. This finding is interesting given that the only indicator of fetal selection we uncover through aggregate data suggests weak indications of fetal selection during the tail end of pregnancy as measured by the stillbirth rate. Linking these results to mechanistic predictions regarding the association between disruptions during the fetal stage and later-life outcomes is challenging. Males’ higher sensitivity to fetal stress is well documented, and the absence of any strong evidence of sex-specific fetal selection suggests that the influenza pandemic did not cause a stronger selection process among male fetuses. The results, however, suggest that influenza exposure was severe enough to cause scarring. Perhaps males’ greater sensitivity became manifest as more significant scarring.

Previous research using individual-level data has generally found negative effects of fetal exposure to the 1918 pandemic on socioeconomic outcomes (Almond 2006; Lin and Liu 2014; Neelsen and Stratmann 2012). The one exception that we are aware of is a study on Sweden by Richter and Robling (2013), which found a positive income effect. A subsequent study that used a different sample and specification, however, found negative effects (Richter and Robling 2015). For males, for whom information on income and occupation is a more reliable indicator of their socioeconomic attainment than for females, we find that those who were exposed to the influenza pandemic *in utero* did not suffer from lower earnings in late adulthood compared with cohorts born five years before or after. If anything, the results show that individuals who were born shortly after the pandemic experienced *higher* earnings at age 55. This effect, however, is not limited to those experiencing fetal exposure but also holds for those exposed in the first 18 months of life. We find analogous results for SES as measured by occupational attainment. This finding could reflect the slightly increased share of high-SES fathers among affected cohorts, where the intergenerational transmission of socioeconomic resources outweighs the effects of an eventual health penalty due to exposure to the pandemic. However, because fetal exposure to the pandemic occurred in a much shorter period than the aforementioned change in parental SES, effects of fetal programming may still be identifiable. Although some cohorts exposed *in utero* did not enjoy the income and occupational attainment advantages of the surrounding birth cohorts—possibly consistent with negative effects of the flu outweighing the positive effects of the change in social gradient toward higher SES for children—the effects are not estimated with sufficient precision to allow us to draw any conclusive conclusions. Furthermore, results for socioeconomic outcomes are not robust to alternative specifications. Consequently, the empirical evidence supporting a causal link between Spanish flu exposure and later-life socioeconomic attainment is weak at best.

We attempt to quantify the extent to which the socioeconomic advantage of the 1918 and 1919 birth cohorts is a realistic result of this socioeconomic composition of fathers. The elevated share of high-SES fathers among the 1918–1919 birth cohorts translates to roughly a 3 % increase for these cohorts compared with the preceding birth cohorts. In a similar exercise on the results from the multivariate analysis, we compare the increase in the predicted CAMSIS score of the groups and find an increase of no more than about 1 % among the 1918–1919 birth cohorts compared with surrounding cohorts. This finding is roughly in line with contemporary estimates of intergenerational earnings elasticities, although the measurements of SES are not identical. Nevertheless, this finding strengthens the belief that the counterfactual outcomes (i.e., if the change in fathers’ socioeconomic composition had not occurred) for the groups exposed in infancy and *in utero* would have pointed to an absence of relationship between influenza exposure and later-life socioeconomic outcomes.

Why was the change in the socioeconomic composition of the 1918–1919 birth cohorts significant enough to (at least partly) offset the negative long-term consequences of influenza exposure for examining socioeconomic outcomes but not for the health outcomes? The intergenerational transmission of SES is arguably one of the most researched topics in the social sciences, offering results that are robust across space and time. The transmission of health is considerably less well studied, but previous studies nevertheless have pointed toward a weaker but tangible process. Consequently, to the extent that the children of high-SES fathers enjoyed a socioeconomic as well as health advantage, the results remain difficult to reconcile with a vast literature of intergenerational processes. There are, however, reasons to believe that this point of departure is flawed. Recent research from several contexts has suggested that health differences by SES, which we take for granted today but in fact is a relatively new phenomenon, in Sweden emerged only well after World War II (Bengtsson and Dribe 2011; Bengtsson and van Poppel 2011). Thus, our results do not contradict the existence a strong process of intergenerational transmission of both SES and health. Instead, our findings are consistent with such a narrative but one in which the health stock being transmitted across generations is not systematically positively related to SES. Furthermore, the moderate nature of observed health effects increase the plausibility that affected cohorts were able to participate in the labor market, thus allowing affected cohorts to enjoy their advantaged socioeconomic position throughout the life course.

## Electronic supplementary material

ESM 1 (PDF 801 kb)

## Footnotes

1 In their analysis of the short-term effects of the Spanish flu on three indicators of economic outcomes in Sweden, Karlsson et al. (2014) combined the annual influenza mortality rate with monthly morbidity rates at the county level.

2 The group of well-off fathers is defined as consisting of landlords and yeomen within farming, sharecroppers, foresters, factory owners and engineers, merchants, higher and lower white-collar workers (in 1920 and earlier), executives (in 1924 and after), civil servants, military officers, priests, teachers, journalists, and a group labeled as capitalists.

3 Results were obtained through Cox proportional hazards regression analyses on all individuals belonging to the 1912–1924 birth cohorts. Estimating the hazard of dying using the individual’s age at death as the duration variable, models control only for the quarter and year of birth.

4 We use a Wald test in Stata to test point estimate equality.
