# Supplementary material for: The Long-Lasting Influenza: The Impact of Fetal Stress During the 1918 Influenza Pandemic on Socioeconomic Attainment and Health in Sweden, 1968–2012
Source: Demography. 2019 Jul 19;56(4):1389–425. doi: 10.1007/s13524-019-00799-x (PMC6667423; doi:10.1007/s13524-019-00799-x)
Supplement: Supplementary file 1 — (PDF 801 kb) [file 13524_2019_799_MOESM1_ESM.pdf]

Appendix

Table A1: Sample means. Standard deviation within brackets.

	Occupational status in 1970	Income in 1970	Hospitalization, age 54-87		Mortality, age 54-87	
	Men	Men	Men	Women	Men	Women
Camsis	47.4					
	[14.6]					
Income (1000's)		34.9				
		[28.1]				
Hospitalization during current year (%)			19.1	16.6		
Days of hospitalization during current year (%):						
0 days			80.9	83.4		
1-5 days			7.5	6.1		
6-10 days			3.9	3.5		
11-20 days			3.4	3.2		
21-30 days			1.6	1.4		
31 days or more			2.7	2.5		
Days of hospitalization during current year			3.2	2.9		
			[13.8]	[13.1]		
Mortality, all cause (%)					78.0	61.0
Mortality, cancer (%)					21.5	17.2
Mortality, cardiovascular (%)					43.6	32.1
Mortality, respiratory (%)					7.2	5.6
Mortality, others (%)					5.8	6.1

Birth year	1919.6	1919.5	1919.8	1919.8	1919.5	1919.5
	[3.4]	[3.4]	[3.4]	[3.4]	[3.4]	[3.4]
Exposure trimester 1 (%)	4.2	4.1	4.2	4.2	4.1	4.2
Exposure trimester 2 (%)	4.1	4.1	4.0	4.1	4.1	4.1
Exposure trimester 3 (%)	3.6	3.6	3.6	3.6	3.6	3.6
Individuals	420 985	450 245	423 748	458 488	442 521	471 547
Observations	420 985	450 245	9 077 746	11 281 861	442 521	471 547

Figure A1: Variation in timing and influenza intensity by selected Swedish counties. Crude Mortality Rate (per 1,000), by month

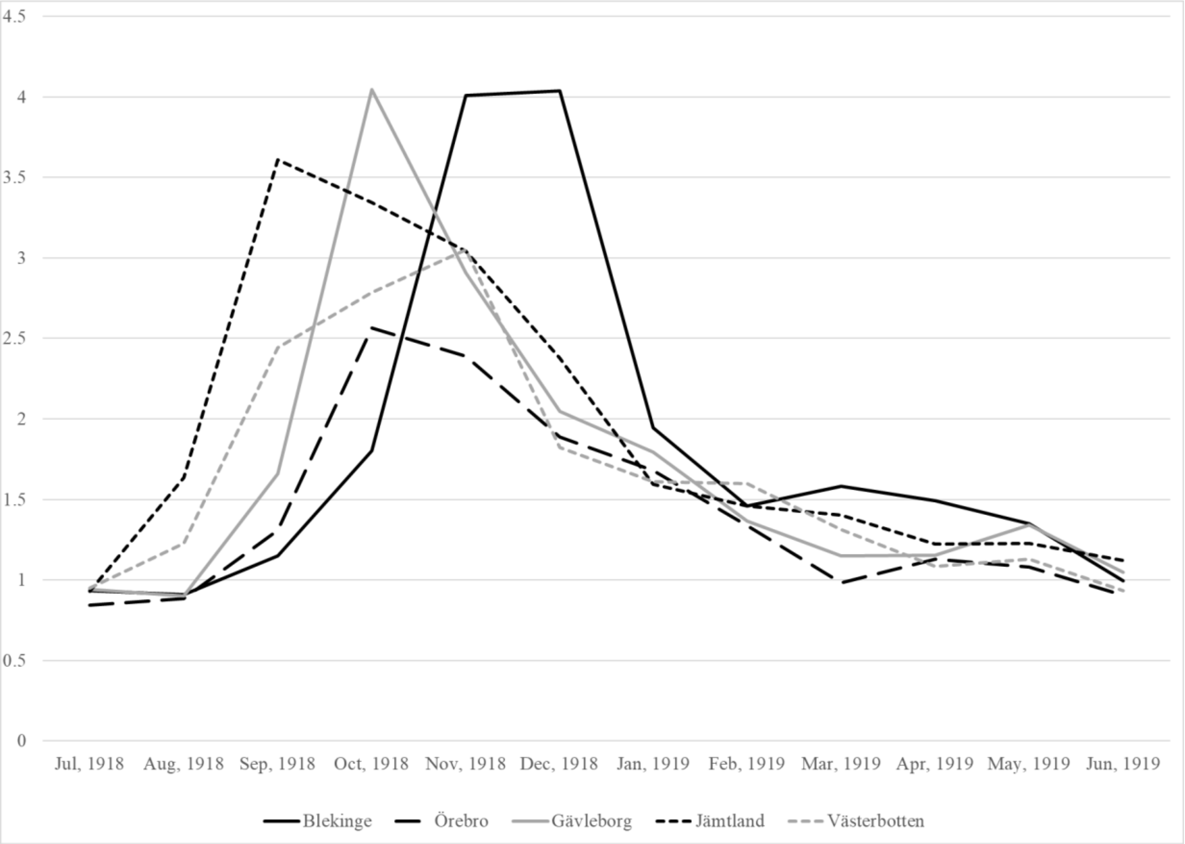

Source: Befolkningsrörelsen, 1918-1920

Figure A2. Influenza morbidity rate by age at different work places in Sweden.

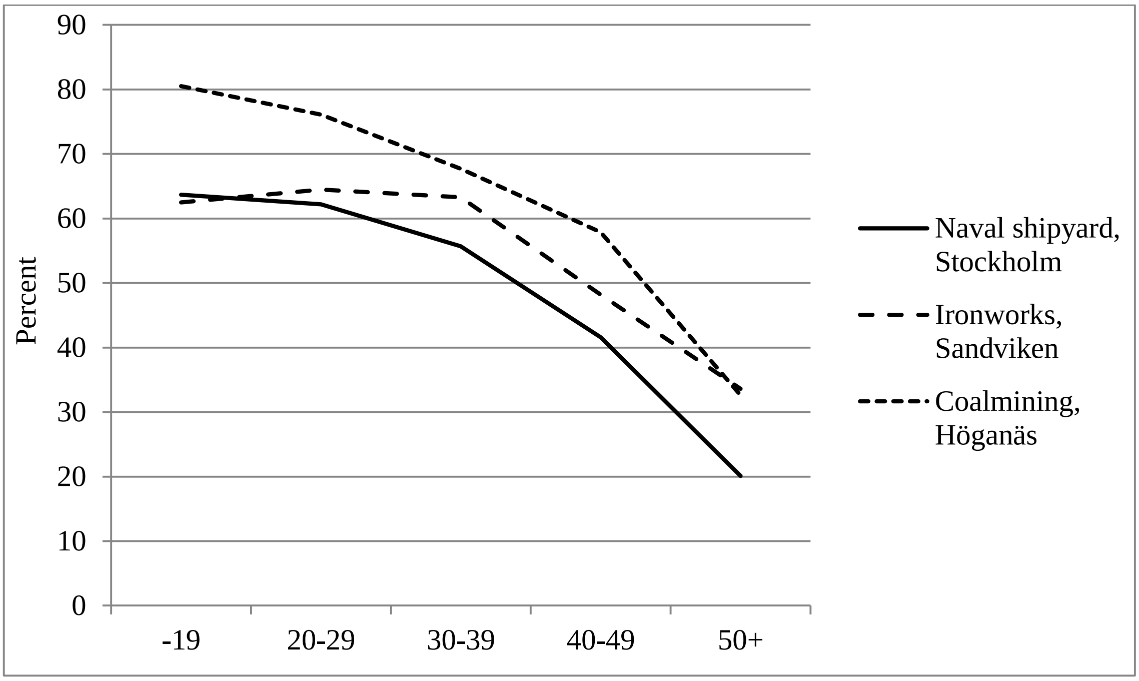

Sources: Alling 1919, Gibson 1919, Widstrand 1918.

Figure A3: Cumulative distribution of morbidity and mortality due to the Spanish flu from July 1818 to June 1919.

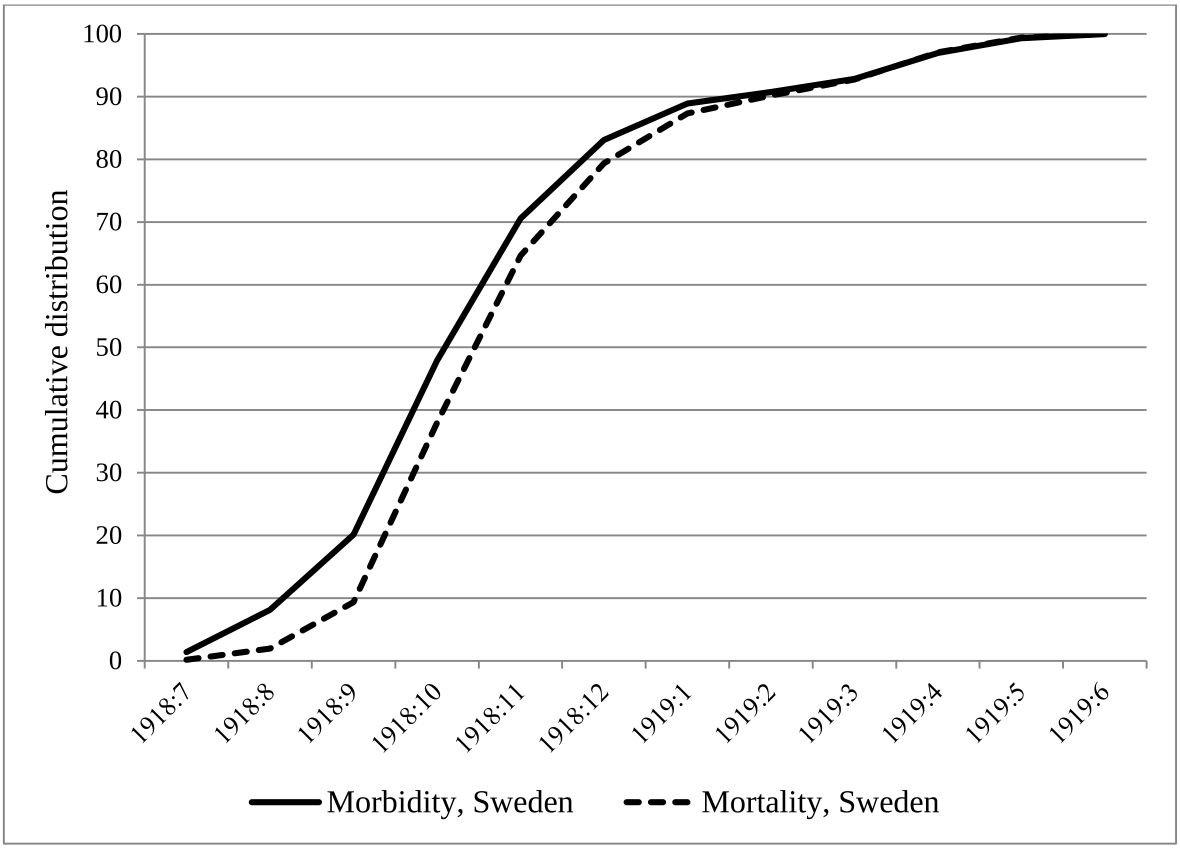

Sources: Lindhagen 1926, Allmän hälso- och sjukvård år 1918 and 1919, for Sweden

Figure A4. Influenza and pneumonia mortality in 1918 by age and sex, Sweden.

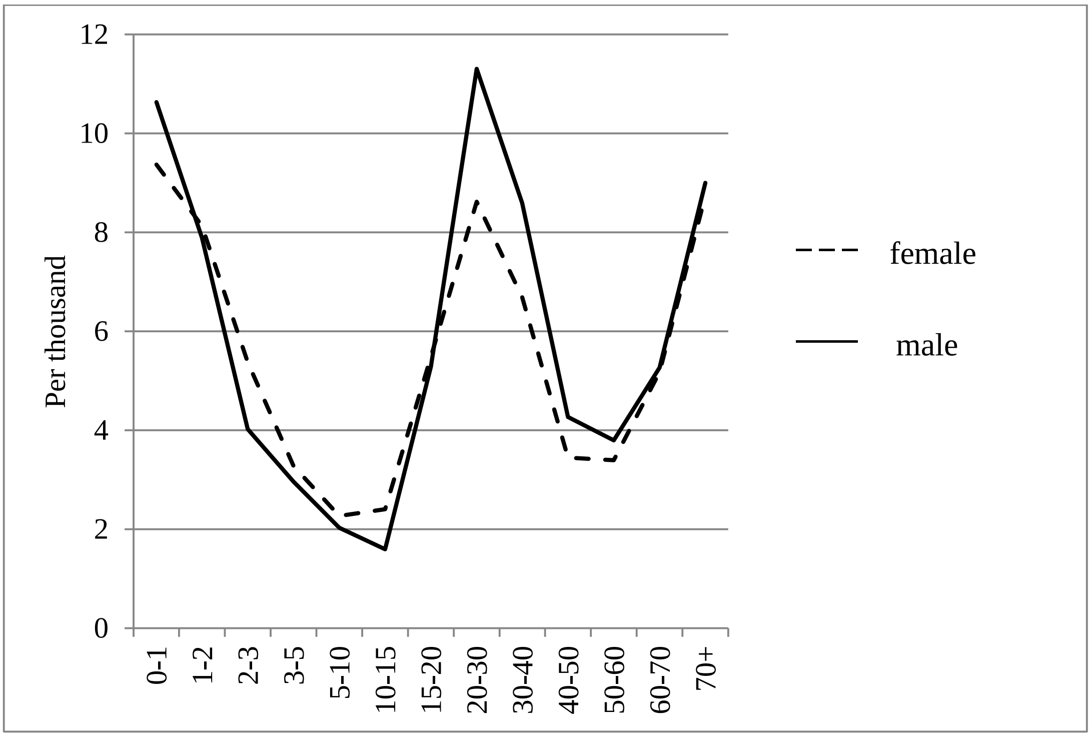

Source: Befolkningsrörelsen 1918.

Figure A5. All-cause mortality by age and sex in 1918 relative to 1917, Sweden.

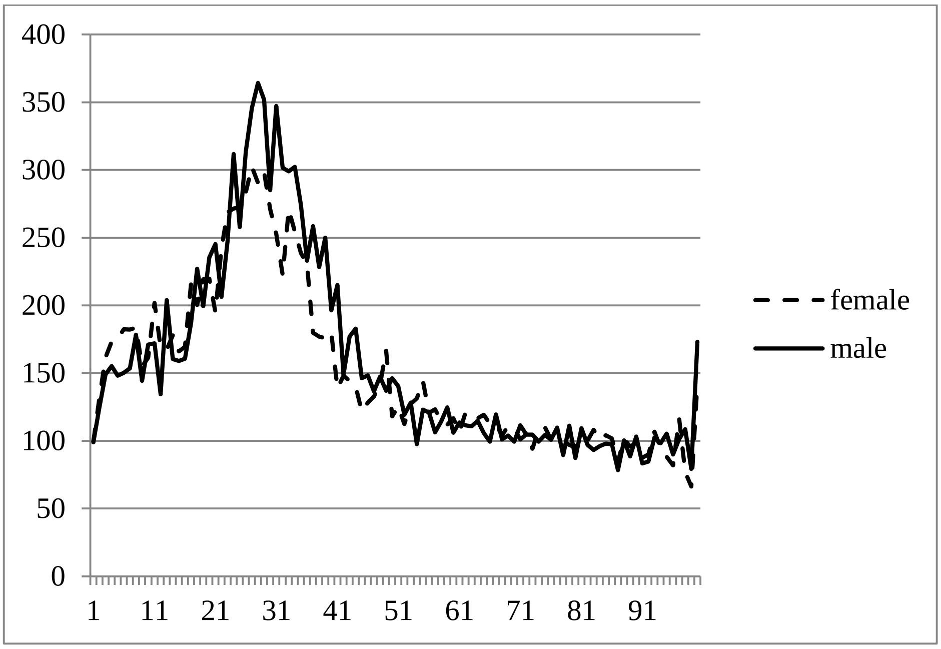

Source: Befolkningsrörelsen 1917, 1918.

Figure A6. Monthly number of live births in Sweden, by conception cohort (nine months before birth), 1914-1925. The shaded vertical bars represent the period October-December 1918.

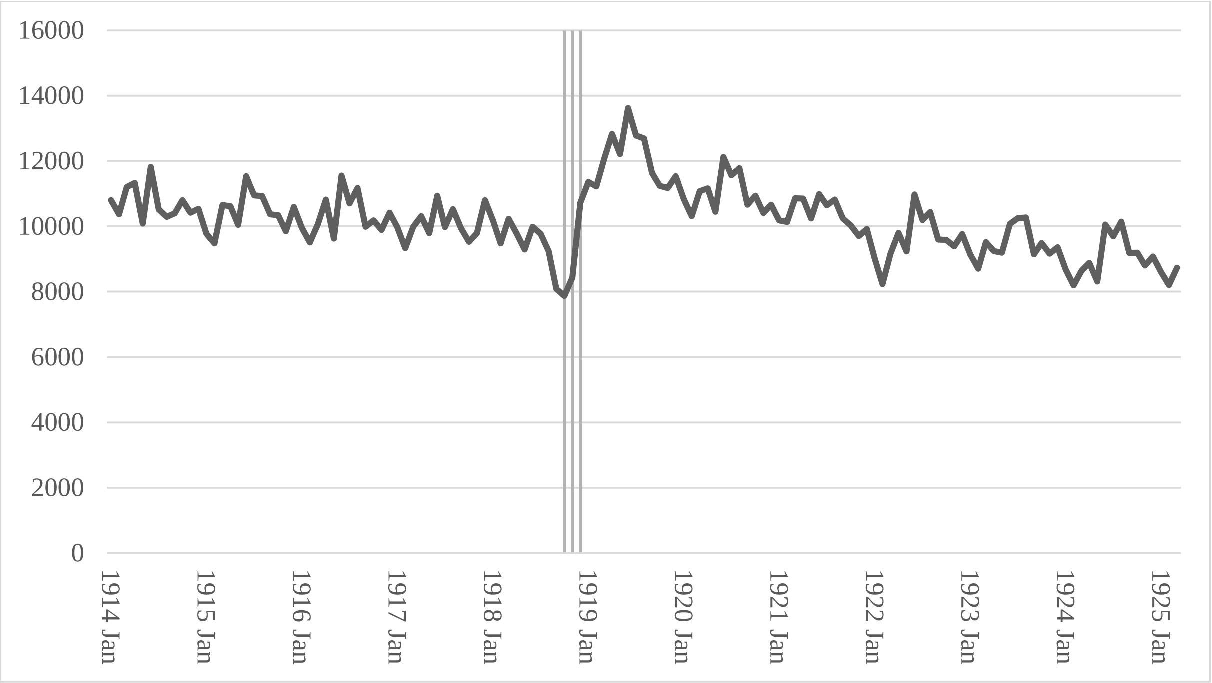

Source: Befolkningsrörelsen 1914-1926

Figure A7. Percentage of high SES fathers and fathers under the age of 30, 1911-1930, Sweden

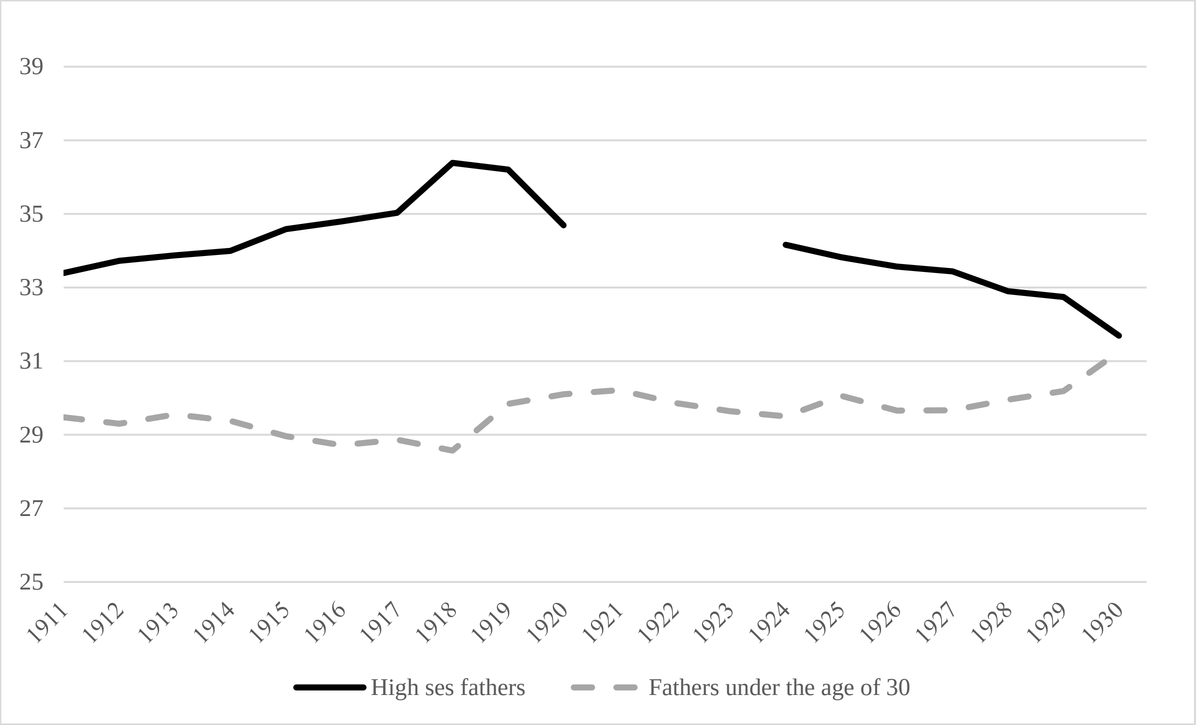

Source: Befolkningsrörelsen 1911-1930

Figure A8. Sex ratio at birth, by month, Sweden 1914-1925. The shaded vertical bars represent the period October-December 1918. The shaded area represents the main cohorts exposed to the influenza pandemic during trimester one.

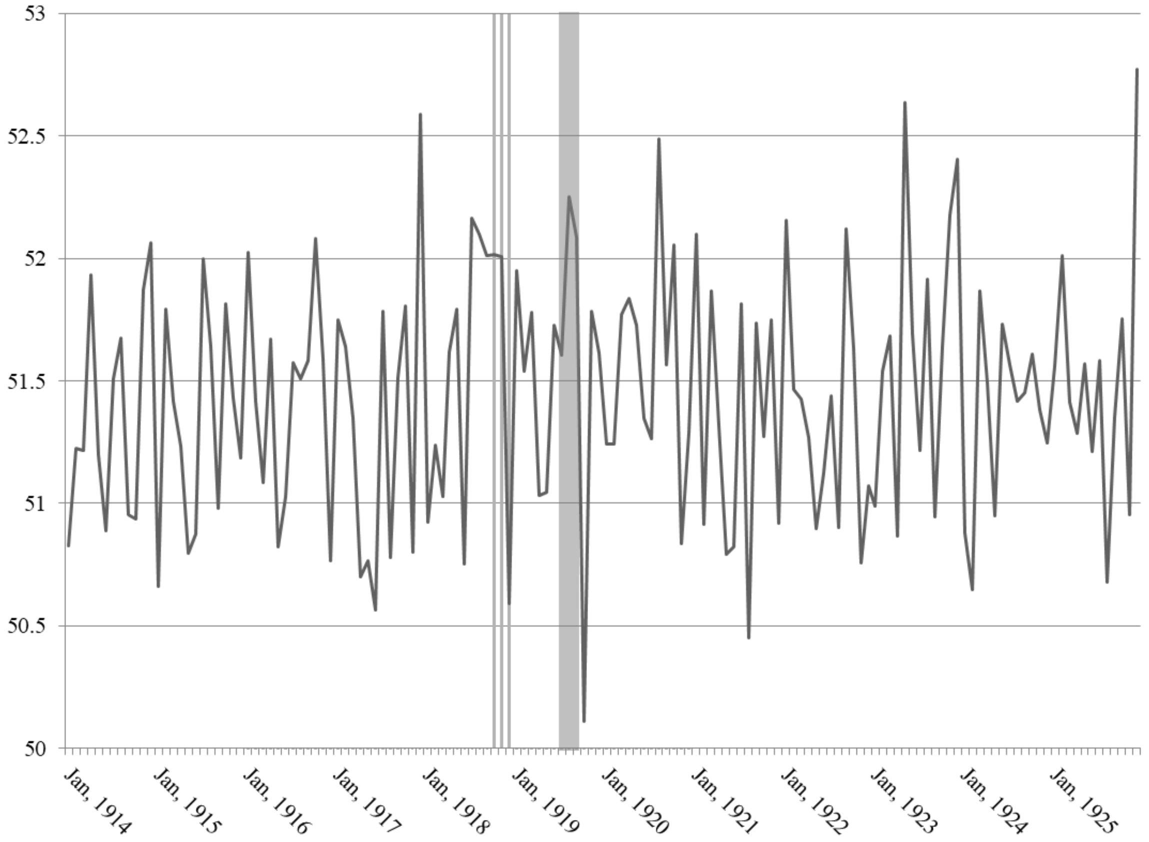

Source: Befolkningsrörelsen 1914-1925.

Figure A9a. Age specific mortality, age 0-1, by birth quarter 1912-1924. Hazard ratios obtained from Cox proportional hazards regression models. Reference category, Q1 1919.

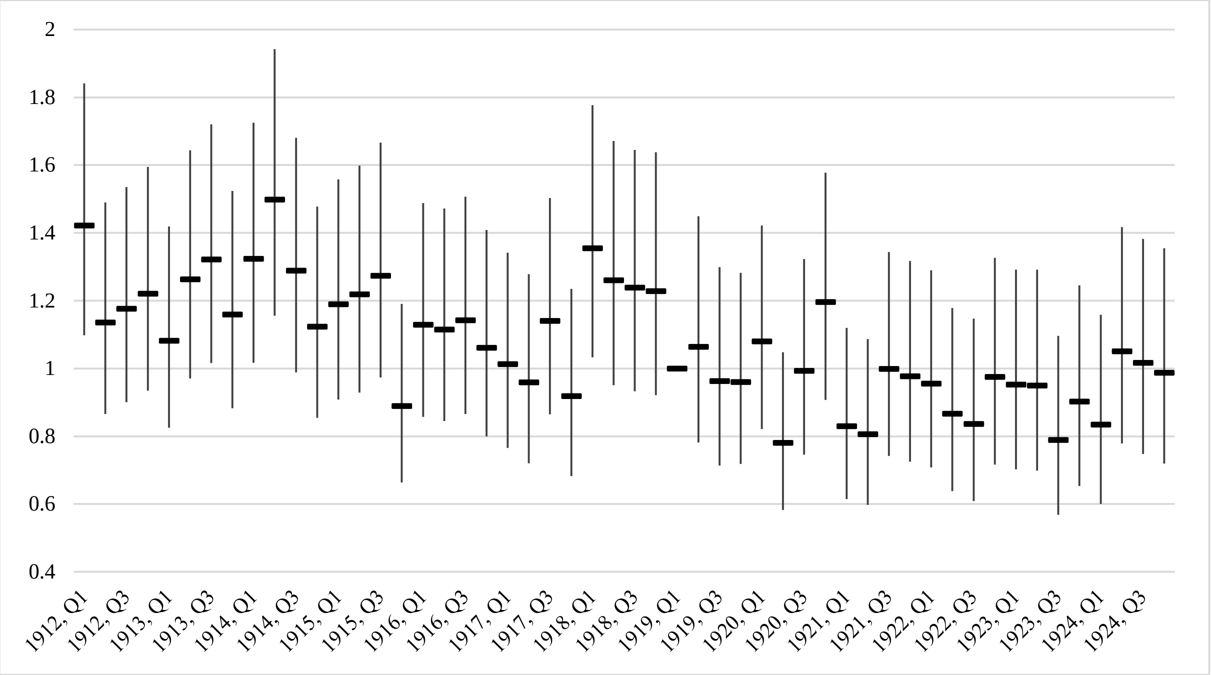

Source: Sveriges släktforskarförbund. Sveriges dödbok, 1901-2013.

Figure A9b. Age specific mortality, age 1-5, by birth quarter 1912-1924. Hazard ratios obtained from Cox proportional hazards regression models. Reference category, Q1 1919.

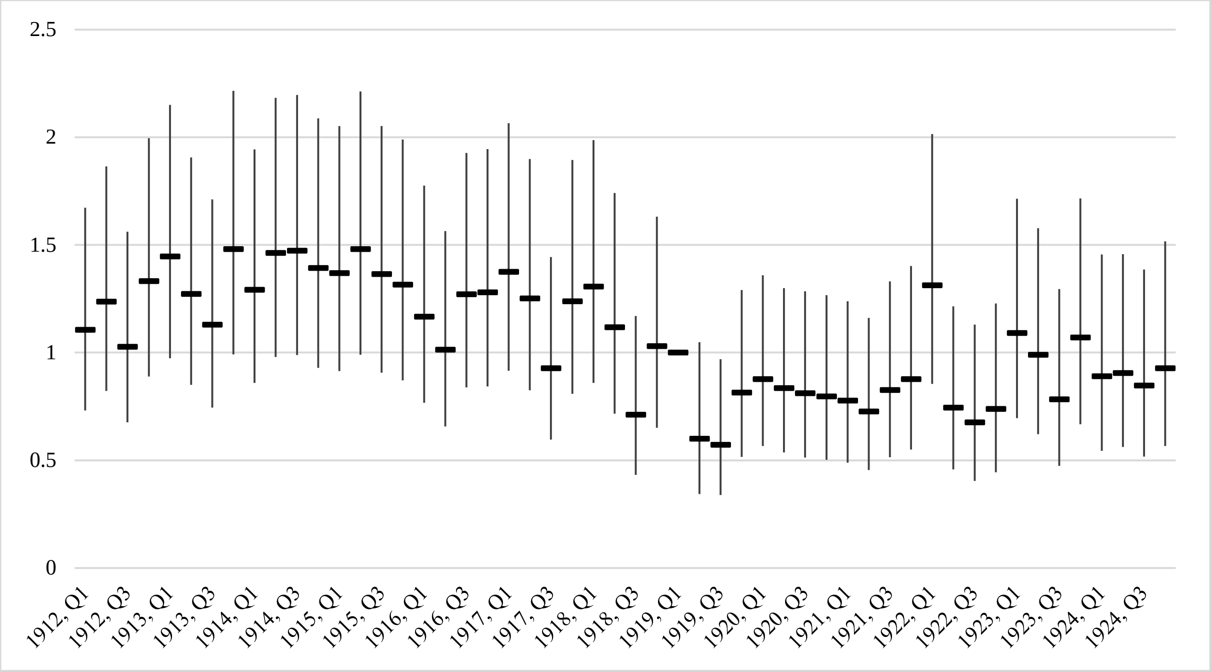

Source: Sveriges släktforskarförbund. Sveriges dödbok, 1901-2013.

Figure A9c. Age specific mortality, age 5-10, by birth quarter 1912-1924. Hazard ratios obtained from Cox proportional hazards regression models. Reference category, Q1 1919.

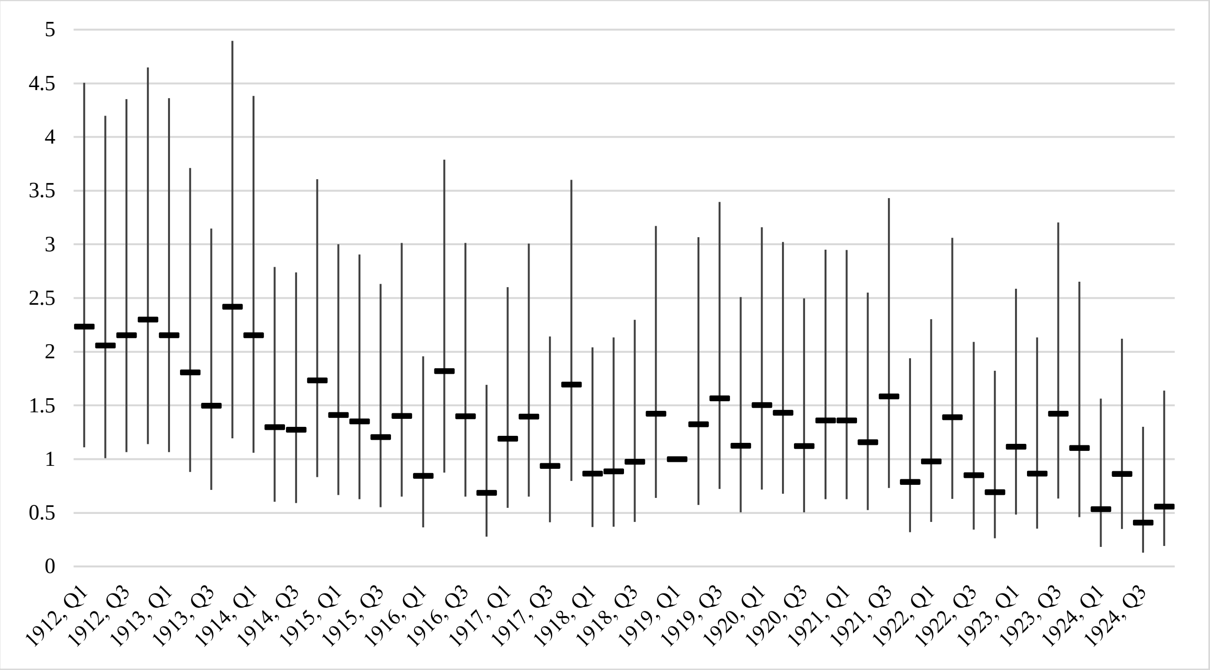

Source: Sveriges släktforskarförbund. Sveriges dödbok, 1901-2013.

Figure A9d. Age specific mortality, age 10-15, by birth quarter 1912-1924. Hazard ratios obtained from Cox proportional hazards regression models. Reference category, Q1 1919.

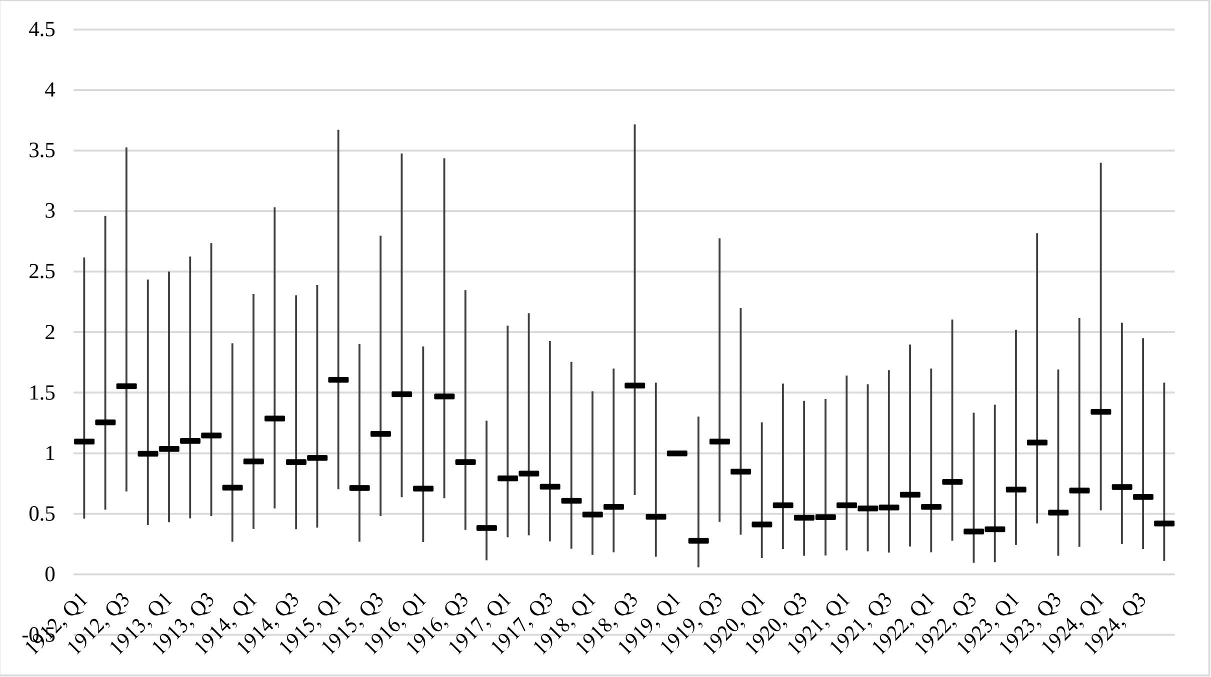

Source: Sveriges släktforskarförbund. Sveriges dödbok, 1901-2013.

Figure A10. Influenza exposure periods, by county.

County			1918				1919			Max relative CDR
	Aug	Sep	Okt	Nov	Dec	Jan	Feb	Mar	Apr	
Stockholm										304
Uppsala										335
Södermanland										389
Östergötland										346
Jönköping										276
Kronoberg										319
Kalmar										310
Gotland										506
Blekinge										399
Kristianstad										294
Malmöhus										439
Halland										249
Göteborgs och Bohus										399
Älvsborgs										466
Skaraborgs										273
Värmland										256
Örebro										289
Västmanland										292
Dalarna										438
Gävleborg										507
Västernorrland										394
Jämtland										454
Västerbotten										259
Norrbotten										297
Share of mid-1918 population exposed	0%	10%	99%	100%	97%	61%	0%	0%	0%	-
Share exposed to high mortality	0%	2%	24%	25%	25%	15%	0%	0%	0%	-
Share exposed to low mortality	0%	3%	40%	32%	29%	22%	0%	0%	0%	-

Notes: Influenza period is defined as occurring from the month when the CDR exceeds 1.75, and until it descends below this threshold. Relative CDR is calculated as the CDR in county *i* and month *t*, divided by the CDR in county *i* and month *t-12*. High mortality counties are defined as having a maximum relative CDR exceeding 400 %, whereas low mortality counties are defined as having a maximum relative CDR not exceeding 300 %.

Legend: Low mortality county; High mortality; Medium mortality

Source: Befolkningsrörelsen 1917-1919.

Figure A11. Mean days of annual hospitalization between the age 54 and 87, by birth year and quarter. The shaded vertical bar represents individuals born during Q1, 1919.

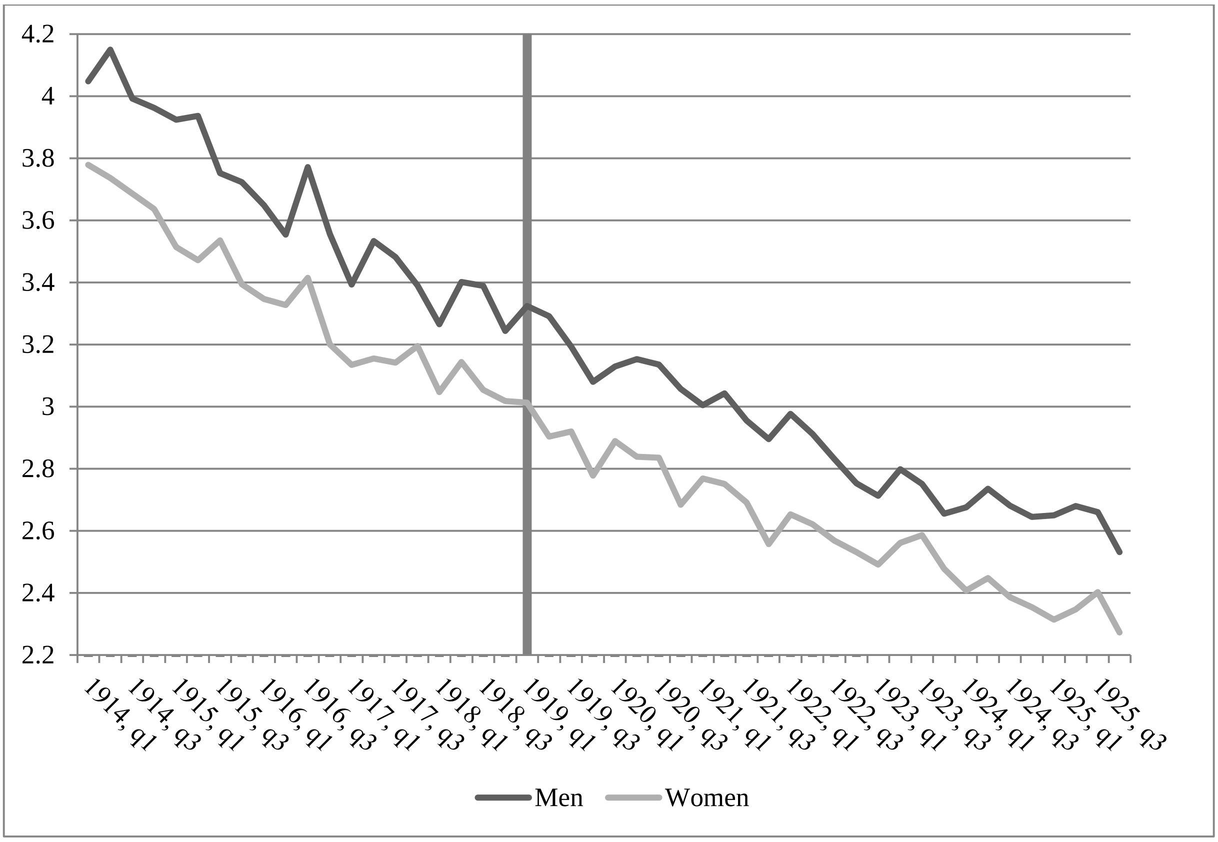

Source: Swedish Interdisciplinary Panel.
